# Evidence for a Potential Context-Dependent Dual Role of Eb Peptide of IGF-1Eb in Cancer Cell Survival and Adaptation to UV Stress

**DOI:** 10.3390/ijms27114793

**Published:** 2026-05-26

**Authors:** Amalia Kotsifaki, Georgia Kalouda, Efthymios Karalexis, Sylvia Raftopoulou, Nektarios Alevizopoulos, Athanasios Armakolas

**Affiliations:** Physiology Laboratory, Medical School, National and Kapodistrian University of Athens, 11527 Athens, Greece; amkotsifaki@med.uoa.gr (A.K.); gkalouda@med.uoa.gr (G.K.); euthimiskaralexis12@gmail.com (E.K.); sraftopoulou@med.uoa.gr (S.R.); nalevizopoulos@gmail.com (N.A.)

**Keywords:** IGF-1Eb, UV stress, cancer cell survival, Eb-peptide, stress adaptation, apoptosis, breast cancer, prostate cancer, liver cancer, potential target

## Abstract

The IGF-1Eb isoform has been proposed as a stress-responsive variant of IGF-1, yet its significance in cancer remains unclear. This investigation aims to clarify its role across breast, prostate and liver cancer cell lines and determine whether its loss or supplementation is associated with alterations in cellular behavior and stress adaptation. Eb expression was modulated through targeted silencing and exogenous peptide addition. Cellular responses were evaluated under normal conditions and UV stress using proliferation, viability and rescue experiments, wound healing, immunofluorescence for Eb-knockdown confirmation, qRT-PCR, Annexin V/PI apoptosis, and PI cell-cycle evaluation. Across six cancer cell lines, Eb peptide given before UV stress was associated with partial protective effects, whereas post-UV treatment was associated with improved recovery and partial restoration of proliferative capacity. The rescue effect differed by cell type, with prostate and breast cells showing the strongest responses and liver-derived lines displaying more modest improvements. Eb knockdown revealed clear cell-type-specific dependencies. PC3 cells showed markedly reduced proliferation (*p* < 0.01) and sharply decreased post-UV viability (*p* < 0.0001). HepG2 cells maintained higher growth without UV but displayed reduced recovery following UV exposure, whereas MDA-MB-231 exhibited elevated apoptosis (*p* < 0.05) with limited additional UV sensitivity. Eb peptide may exert a dual, timing-dependent role, supporting protection before UV damage and facilitating recovery-associated responses afterward, with its impact differing across cell lines.

## 1. Introduction

The insulin-like growth factor (IGF) system represents a fundamental regulatory network in mammalian biology, governing processes such as growth, differentiation, metabolism, and cell survival [1]. Its ligands (IGF-1 and IGF-2), receptors (IGF-1R and IGF-2R), and IGF-binding proteins (IGFBPs) coordinate endocrine, paracrine, and autocrine signaling, exerting wide-ranging physiological effects [2]. Structurally similar to insulin, IGF-1 interacts primarily with IGF-1R and, with lower affinity, with the insulin receptor, thereby linking metabolic and mitogenic functions [3,4]. The human IGF1 gene, located on chromosome 12, undergoes alternative splicing to generate multiple transcript variants, namely IGF-1Ea, IGF-1Eb, and IGF-1Ec [5]. These isoforms differ mainly in their E-peptide extensions, which influence post-translational processing and may confer distinct biological activities, particularly under pathological conditions [6]. Specifically, alternative splicing of exons 4–6 gives rise to the distinct E-peptides: IGF-1Ea (exons 4 and 6), IGF-1Eb (exons 4 and 5), and IGF-1Ec (exon 4 with an alternative splice acceptor site within exon 6). This post-transcriptional diversification may contribute to context-dependent modulation of IGF-1 signaling, which may be contextually modulated, particularly under conditions of cellular stress or tissue remodeling [7].

Among the IGF-1 isoforms, IGF-1Ea is the most abundant, predominantly expressed in the liver, where it maintains circulating IGF-1 levels [5]. It mediates the canonical functions of IGF-1, including systemic growth hormone-dependent regulation of metabolism and somatic growth [8]. Beyond its physiological roles, IGF-1Ea has been implicated in cancer, where it has been associated with enhanced proliferation and survival across various malignancies. In contrast, IGF-1Ec has gained considerable attention in cancer biology due to its distinct mitogenic and migratory properties [6]. Ec-derived peptides (PEc) have been shown to promote aggressive phenotypes, including EMT-driven invasion, metastasis, and therapy resistance, particularly in prostate cancer, where IGF-1Ec expression is minimal in healthy tissue, but markedly increased in advanced disease, correlating with tumor grade and progression [9,10,11]. Isoform-specific targeting of the PEc has been reported to suppress these malignant features both in vitro and in vivo [6,9,10,11]. Nevertheless, not all IGF-1 splice variants have been equally explored, and important gaps remain regarding the contribution of other isoforms to cancer-associated stress adaptation.

In particular, while IGF-1Ea and IGF-1Ec have been extensively characterized in cancer, the functional role of IGF-1Eb remains largely unexplored. Previous work has demonstrated stress-inducible regulation of IGF-1Eb expression and has suggested a possible role for the Eb peptide in tissue repair, cellular adaptation, and survival under adverse conditions [12,13,14,15,16]. However, the functional significance of IGF-1Eb in malignant cells, particularly with respect to cancer cell survival and recovery following genotoxic stress, remains incompletely characterized.

Given the established involvement of the IGF axis in tumor progression and therapy resistance, Breast cancer (BC) represents a particularly relevant context in which to examine isoform-specific stress responses [1,13]. Physiologically, IGF-1 plays an important role in mammary gland development; however, in malignancy, its sustained activation promotes proliferation, survival, and therapy resistance through the PI3K/AKT and MAPK pathways [17]. Crosstalk with estrogen receptor (ER) and HER2 signaling may further contribute to tumor aggressiveness [18]. Epidemiological data corroborates these mechanistic observations, linking elevated circulating IGF-1 levels with increased BC risk and demonstrating IGF-1R activation in nearly half of BCs, where it is associated with poor prognosis [1,17,19]. Despite this, isoform-specific contributions to BC stress responses and damage recovery remain poorly defined.

Similar patterns are observed in prostate cancer, one of the most prevalent and lethal malignancies in men [20]. Elevated circulating IGF-1 levels and enhanced IGF-1R activation are consistently associated with increased tumor aggressiveness and poor clinical outcomes [21]. Beyond membrane signaling, nuclear localization of IGF-1R in malignant prostate cells has been shown to influence DNA repair and stress tolerance, highlighting the ability of this pathway to support cell survival under genotoxic stress [20]. Whether distinct IGF-1 isoforms differentially contribute to these stress-adaptive mechanisms remains unclear [20,21].

Building on these insights, the liver offers a distinct framework for investigating IGF-1 isoforms, given its dual role as the principal source of systemic IGF-1 and as a frequent site of malignant transformation [22]. In hepatocellular carcinoma (HCC), IGF-1 drives EMT, invasion, and metastasis through activation of the PI3K/AKT and MAPK signaling cascades, while IGF-2 overexpression and peptide modifications further reinforce oncogenic signaling [22,23]. Epidemiological studies highlight the complexity of IGF biology, as both low and high circulating IGF-1 levels have been associated with adverse clinical outcomes [24]. These observations underscore the importance of tissue- and isoform-specific regulation of IGF-1 signaling in tumor adaptation [25].

Taken together, these findings highlight the broad involvement of IGF-1 signaling as a central driver of cancer hallmarks, including uncontrolled proliferation, migration, angiogenesis, and resistance to apoptosis [26]. Through activation of the PI3K/AKT, MAPK, and JAK/STAT cascades, IGF-1 broadly sustains oncogenic processes across diverse tumor types [27]. However, the limited clinical success of IGF-1R inhibitors suggests that isoform-specific and non-canonical signaling routes could potentially contribute to resistance mechanisms and persistent tumor survival [26]. This realization highlights the need to better understand lesser-studied IGF-1 isoforms that may contribute to cellular stress tolerance independently of classical IGF-1R signaling [28].

Although the oncogenic relevance of IGF-1 signaling has been extensively documented, the isoform-specific contributions remain poorly understood [1,5]. Among these, IGF-1Eb represents a relatively understudied candidate, as existing evidence links its expression to stress conditions, yet its functional role in cancer cell stress adaptation has not been systematically examined. To address this gap, the aim of the present study was to investigate the role of the Eb peptide of IGF-1Eb in cancer cell survival and stress responses. By combining IGF-1Eb knockdown with exogenous Eb supplementation across breast, prostate, and liver cancer cell lines, we sought to test the hypothesis that IGF-1Eb exerts a context- and timing-dependent effect on cellular viability, repair capacity, and proliferative recovery following UV-induced stress.

## 2. Results

### 2.1. Combined EMSA and DRB Assays

**Figure 1:**
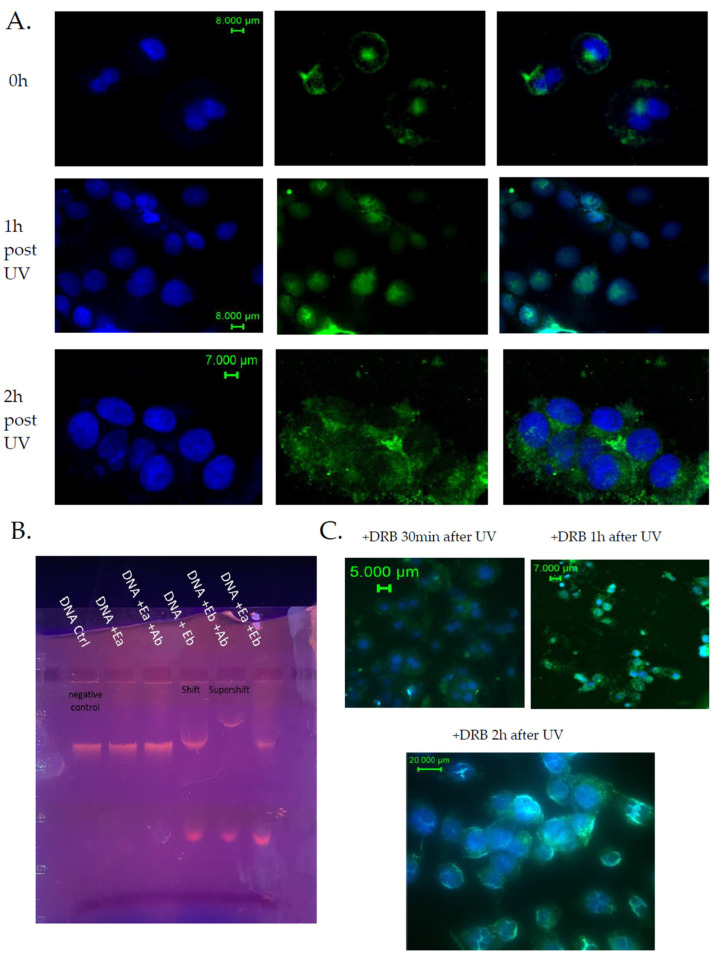
Dynamic localization, DNA-binding activity of Eb peptide following UV treatment and transcriptional blockade with DRB. (**A**) Immunofluorescence analysis of Eb peptide (green) in PC3 prostate cancer cells at 0, 1 and 2 h after UV exposure. At 0 h, Eb peptide is predominantly cytoplasmic. At 1 h, a clear nuclear accumulation is observed. By 2 h, Eb exits the nucleus and shows enhanced cytoplasmic intensity compared with baseline. DAPI marks nuclei (blue). (**B**) Electrophoretic mobility shift assay demonstrating direct DNA binding by recombinant IGF-1Eb. The DNA control shows a single unbound band, whereas addition of IGF-1Eb produces a distinct mobility shift consistent with formation of a DNA–protein complex. Inclusion of an anti-IGF-1Eb antibody results in a supershift, confirming the specificity of the interaction. In contrast, IGF-1Ea does not alter DNA mobility, indicating a lack of detectable DNA-binding activity under identical conditions. (**C**) Immunofluorescence analysis of Eb localization in PC3 cells following transcriptional inhibition with DRB and subsequent UV exposure. In DRB-treated cells, Eb (green) remains predominantly nuclear, as shown by co-localization with DAPI (blue). Nuclear retention is preserved after 30 min or 1 h UV irradiation in the presence of DRB, as well as after prolonged UV treatment with DRB, demonstrating that Eb peptide maintains stable nuclear localization even under transcriptional blockade and acute UV stress.

**Figure A1:**
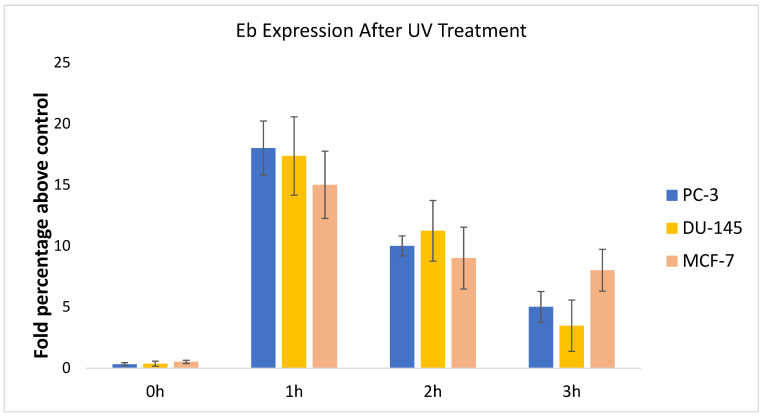
UV-induced changes in Eb expression in PC3, DU145 and Mcf-7 cells. PCR analysis revealed very low expression of Eb at 0 h for all three cell lines, followed by a significant increase after UV exposure. The maximal expression was observed 1 h after UV exposure in all cell lines, and then gradually declined by 3 h. Expression levels were normalized to GAPDH. All data are presented as mean ± SEM (n = 4).

Immunofluorescence imaging showed a distinct temporal pattern in Eb distribution after UV exposure. At baseline (0 h), Eb was mainly cytoplasmic. One hour after irradiation, Eb peptide accumulated within the nucleus, indicating a rapid stress-responsive translocation. By 2 h, the protein had redistributed back to the cytoplasm, where its signal was more intense than at 0 h, possibly reflecting increased cytoplasmic abundance or stabilization (Figure 1A). To validate the immunofluorescence observations and determine whether the differences in Eb localization were accompanied by altered Eb expression, Eb mRNA levels were analyzed by PCR in three cell lines (PC3, DU145 and Mcf-7), at 0–2 and 3 h after UV radiation. Consistent with what was observed in the immunofluorescence experiment, Eb expression levels were minimal at 0 h, followed by a significant increase after UV exposure, reaching a peak at 1 h and then gradually declining again by 3 h (PC3 Eb expression: 0.32 ± 0.12 (0 h) vs. 18 ± 2.1 (1 h) vs. 10 ± 0.8 (2 h) vs. 5 ± 1.25 (3 h)) (Appendix A, Figure A1). Agarose gel electrophoresis revealed a clear mobility shift when DNA was incubated with recombinant IGF-1Eb, indicating the formation of a DNA–protein complex. The introduction of a specific anti-IGF-1Eb antibody produced an additional supershift, confirming the specificity of this interaction. In contrast, IGF-1Ea failed to induce any detectable shift under the same conditions. These results suggest that IGF-1Eb is capable of directly or stably associating with DNA, supporting the possibility of a nuclear functional role distinct from that of the classical secreted IGF-1Ea isoform (Figure 1B). To determine whether Eb is engaged in transcription-related processes, PC3 cells were treated with the RNA polymerase II inhibitor DRB prior to UV exposure. Immunofluorescence imaging showed that IGF-1Eb remained predominantly localized within the nucleus both before and after DRB treatment. The sustained nuclear retention of Eb despite transcriptional inhibition indicates that this isoform does not rely on active transcription for its localization and does not behave as a conventional transcription factor (Figure 1C).

### 2.2. Effects of Eb Exogenous Addition on Cell Proliferation and Viability

**Figure 2:**
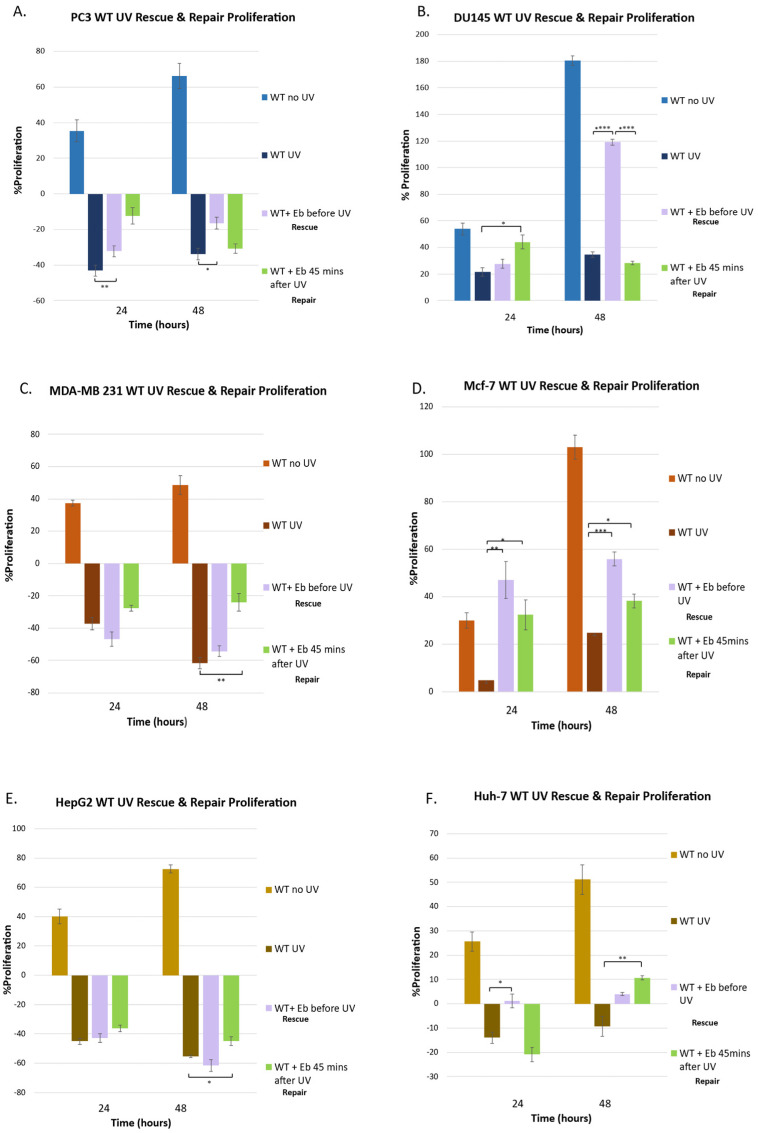
Exogenous Eb peptide enhances proliferative recovery after UV-induced damage across prostate, breast and liver cancer cell lines. (**A**,**B**) Proliferation percentages of prostate cell lines (PC3, DU145) under control conditions, following UV radiation, and after exogenous Eb administration either before or after UV treatment. (**C**,**D**) Proliferation percentages of breast cancer cell lines (MDA-MB-231, Mcf-7) under control conditions, following UV radiation, and after exogenous Eb administration either before or after UV treatment. (**E**,**F**) Proliferation percentages of liver cancer cell lines (HepG2, Huh-7) under control conditions, following UV radiation, and after exogenous Eb administration either before or after UV treatment. All data presented corresponds to WT cells. Bars represent mean ± SEM from three independent biological replicates. Asterisks indicate statistically significant differences based on Tukey’s HSD post hoc test (* *p* < 0.05, ** *p* ≤ 0.01, *** *p* < 0.001, **** *p* ≤ 0.0001).

Proliferation analyses were performed across prostate, breast, and liver cancer cell lines to determine whether exogenous Eb supplementation modulates cellular recovery following UV stress. For all datasets, statistical evaluation was performed using one-way ANOVA, comparing WT + UV, WT + UV + Eb (before UV) and WT + UV + Eb (after UV) conditions. More specifically, in the prostate cancer cell lines PC3 and DU145, UV irradiation caused a pronounced reduction in proliferation at both 24 h and 48 h (*p* < 0.01). Although PC3 cells showed no early rescue at 24 h, DU145 cells exhibited a modest but significant improvement when Eb was applied immediately after UV (*p* < 0.05, g = 2.4). By 48 h, both cell lines demonstrated a clear time-dependent shift: Eb peptide administered before UV produced a strong and highly significant recovery (*p* < 0.001 to *p* < 0.0001), whereas post-UV treatment exerted either limited or no sustained effect. These findings indicate that prostate cancer cells benefit most from prophylactic Eb exposure, with DU145 displaying additional short-term responsiveness to post-UV treatment (Figure 2A,B).

A similar pattern was observed in the BC cell lines MDA-MB-231 and MCF-7, where UV exposure significantly suppressed proliferation at all time points (*p* < 0.01). In both lines, Eb pretreatment markedly improved recovery relative to UV alone, yielding significant rescue at 24 h and robust enhancement at 48 h (*p* < 0.01 to *p* < 0.001). Post-UV Eb treatment provided a more modest benefit, reaching significance only in selected conditions, mainly in MDA-MB-231 cells. Direct comparison showed that the addition of Eb before UV was significantly superior to post-treatment, particularly at later recovery stages (*p* < 0.05). These outcomes indicate that BC cells respond strongly to Eb-mediated protection when the peptide is present prior to UV-induced damage (Figure 2C,D).

In the liver cancer cell lines HepG2 and Huh-7, UV irradiation again produced substantial antiproliferative effects. Eb peptide pretreatment led to small but measurable improvements at 24 h and modest positive recovery at 48 h. More specifically, at 48 h the effect is more pronounced, with HepG2 cells showing a strong and sustained suppression of proliferation following UV exposure, even after rescue/repair, whereas in Huh-7 cells the repair group displays a partial recovery of proliferative capacity at this time point.

Although the rescue patterns in liver models were less pronounced than those seen in prostate and breast cells, Eb still showed modest protective effects, with modest significance emerging in specific comparisons (*p* < 0.05). Collectively, across all three organ systems, addition of Eb peptide consistently mitigates UV-induced proliferative loss, with the most reliable and significant benefits achieved when administered before UV exposure, supporting a predominantly protective rather than reparative mechanism (Figure 2E,F).

**Figure A2:**
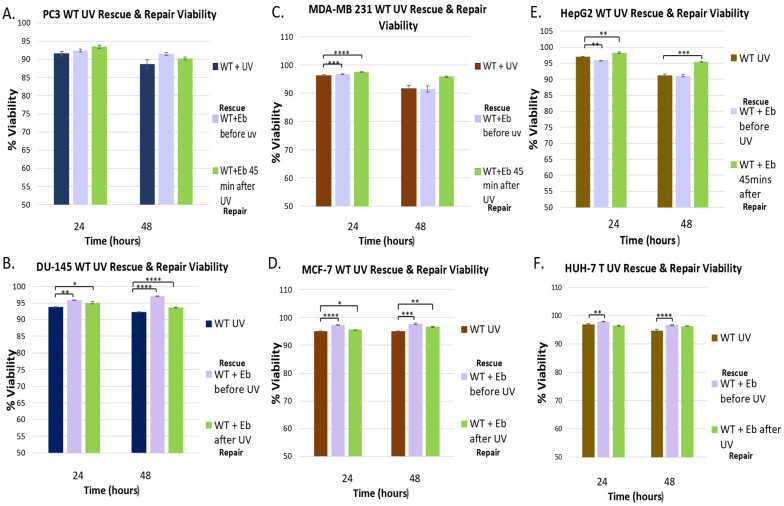
Effects of exogenous Eb peptide on cell viability following UV exposure. Viability responses to UV irradiation and exogenous Eb peptide supplementation varied across the six cancer cell lines examined. (**A**,**B**) In prostate cells, PC3 showed no significant changes under any condition, indicating limited responsiveness to the peptide, whereas DU145 exhibited a modest reduction in viability following UV exposure that was partially reversed by Eb treatment, particularly at 48 h (*p* < 0.0001). (**C**,**D**) In the breast cancer (BC) lines, MDA-MB-231 displayed early responses, with both pre- and post-UV Eb treatment leading to slight increases in viability at 24 h (*p* < 0.001–0.0001), while no significant differences were observed at 48 h. In MCF-7 cells, UV exposure resulted in a slight decrease in viability at 24 h, whereas Eb pretreatment led to a modest but significant increase (*p* < 0.05–0.01), with post-UV treatment showing the highest values at this timepoint. At 48 h, viability remained comparable or slightly reduced under UV, and Eb treatment was associated with increased viability, with pretreatment showing a stronger effect (*p* < 0.001) than post-treatment (*p* < 0.01), despite small absolute differences (1–2%). (**E**,**F**) In liver cancer cell lines, Huh-7 cells showed a slight reduction in viability following UV exposure, while Eb pretreatment was associated with increased viability at both timepoints, reaching stronger significance at 48 h (*p* < 0.0001). Post-UV Eb treatment resulted in intermediate values. Similarly, HepG2 cells exhibited modest UV-associated reductions in viability, with both Eb pre- and post-treatment leading to slightly increased viability at 24 h (*p* < 0.01), while post-UV treatment showed a modest but statistically significant increase at 48 h (*p* < 0.001). Asterisks indicate statistically significant differences based on Tukey’s HSD post-hoc test (* *p* < 0.05, ** *p* ≤ 0.01, *** *p* < 0.001, **** *p* ≤ 0.0001).

In DU145 cells, UV exposure reduced viability relative to WT at both 24 h and 48 h. Eb pretreatment consistently increased viability at both timepoints, with the effect at 48 h showing very strong statistical significance (*p* < 0.0001, g = 7). Eb peptide administered after UV showed a similar upward trend, although less pronounced. In contrast, PC3 cells did not exhibit notable viability changes under any Eb supplementation conditions, suggesting that this line shows limited responsiveness to the peptide in viability assays (Appendix A, Figure A2).

Additionally, in MDA-MB-231 cells, exogenous Eb addition produced measurable effects at 24 h, where pretreatment increased viability (*p* < 0.001, g = 1.5) and post-UV Eb led to a slightly stronger effect (*p* < 0.0001, g = 3.5). By 48 h, however, no Eb condition showed statistical significance, indicating that the peptide’s impact on this line is mainly confined to earlier timepoints. In MCF-7 cells, UV irradiation resulted in a reduction in viability at 24 h, while Eb peptide pretreatment led to a modest but significant increase (*p* < 0.05–0.01, g = 6). Post-UV treatment showed the highest viability at this early stage. At 48 h, UV exposure again reduced viability relative to WT, and Eb modulated this response: pretreatment induced a clear increase (*p* < 0.001, g = 6), while post-UV Eb resulted in a moderate but still significant increase (*p* < 0.01, g = 5). Although the absolute differences between pre- and post-treatment were small (1–2%), they remained statistically significant, suggesting a consistent, albeit biologically modest, effect. In liver cancer cell lines, Huh-7 cells showed a reduction in viability following UV exposure at both timepoints. Eb pretreatment led to a modest increase at 24 h (*p* < 0.01), which became more pronounced by 48 h (*p* < 0.0001, g = 2.1), despite the relatively small magnitude of change (1–2%). Post-UV Eb resulted in intermediate values, without a major deviation from the UV condition. Similarly, HepG2 cells exhibited UV-induced reductions in viability. Both Eb pretreatment and post-treatment resulted in increased viability at 24 h (*p* < 0.01 for both), while at 48 h, post-UV Eb treatment remained significantly elevated (*p* < 0.001, g = 8), indicating that this cell line retains responsiveness to exogenous Eb at later stages (Appendix A, Figure A2).

### 2.3. Effects of Eb Exogenous Addition on Wound Healing Assay

**Figure 3:**
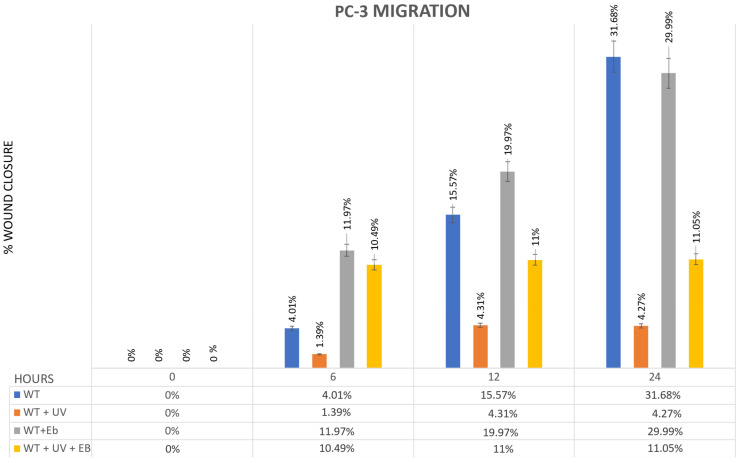
Exogenous Eb peptide enhances PC3 cell migration and partially restores motility under UV-induced stress. Quantification of wound closure expressed as percentage reduction in the wound gap at 6, 12, and 24 h under four conditions: wild-type (WT), WT exposed to UV (WT + UV), WT treated with Eb peptide (WT + Eb), and UV-exposed cells treated with Eb (WT + UV + Eb).

The migratory behavior of PC3 cells was assessed using wound healing assays under control conditions, UV exposure, and exogenous Eb supplementation. WT cells exhibited steady and progressive wound closure, reaching approximately 31–32% by 24 h. UV-irradiated cells showed markedly impaired migration, with closure remaining minimal (~4% at 24 h). Eb treatment enhanced migration under baseline conditions, producing closure levels comparable to WT (~30% at 24 h), and partially restored motility following UV exposure, increasing closure to ~11% compared with UV alone. Collectively, these findings suggest that Eb may support PC3 cell migration and that it provides partial protection against UV-induced migratory deficits (Figure 3).

### 2.4. Immunofluorescence Analysis of Eb Expression in Cancer Cell Lines

**Figure A3:**
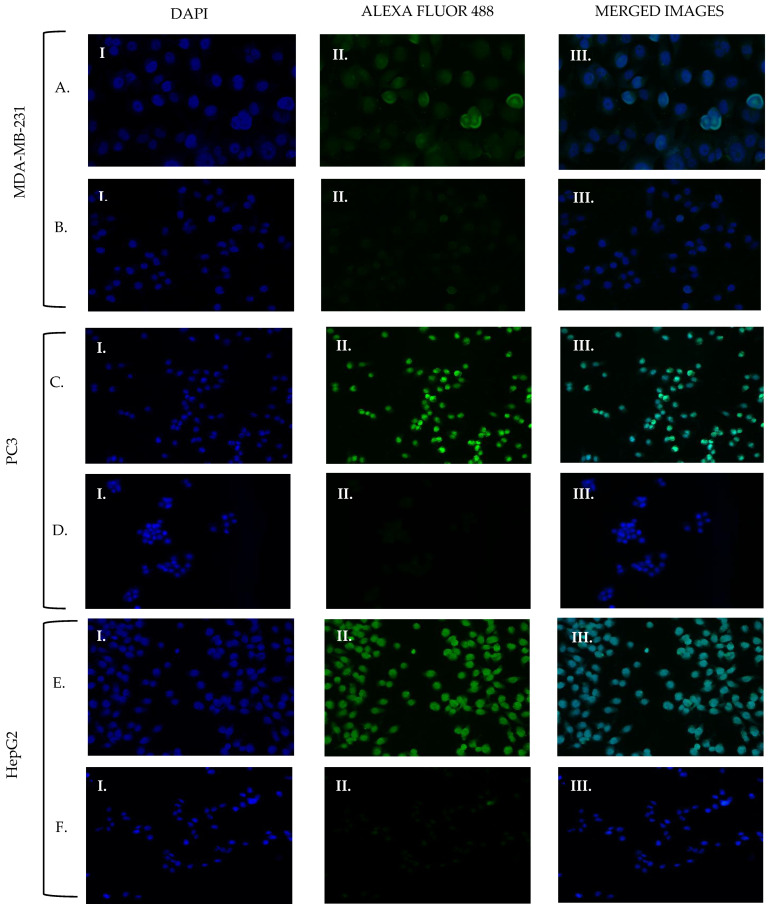
Immunofluorescence analysis of Eb protein expression in control and knockdown cells. Representative images of WT and EbKD cells stained with DAPI (blue) to label nuclei and Alexa Fluor 488 (green) to detect the Eb protein. (**A.I**) MDA-MB-231 control cells stained with DAPI, (**A.II**) Alexa Fluor 488 indicating Eb expression, (**A.III**) merged image. (**B.I**) MDA-MB-231 Eb knockdown cells stained with DAPI, (**B.II**) Alexa Fluor 488 showing reduced signal, (**B.III**) and merged image. (**C.I**) PC3 control cells stained with DAPI, (**C.II**) Alexa Fluor 488 to detect the Eb protein, (**C.III**) merged image. (**D.I**) PC3 Eb knockdown cells stained with DAPI, (**D.II**) Alexa Fluor 488 and (**D.III**) merged image. (**E.I**) HepG2 control cells stained with DAPI, (**E.II**) Alexa Fluor 488 and (**E.III**) merged image. (**F.I**) HepG2 Eb knockdown cells stained with DAPI, (**F.II**) Alexa Fluor 488 and (**F.III**) merged image.

All immunofluorescence experiments were conducted using the EVOS™ M7000 Imaging System (Thermo Fisher Scientific, Waltham, MA, USA) under identical exposure settings to ensure reliable comparison of fluorescence intensity between control and knockdown conditions. Immunofluorescence analysis revealed a marked reduction in Eb protein expression in all cell lines following targeted knockdown. Control cells displayed strong cytoplasmic green fluorescence, indicative of abundant Eb expression, while EbKD cells displayed markedly attenuated signal intensity. Nuclear counterstaining with DAPI confirmed comparable cell density and morphology across conditions. These findings confirm successful silencing of Eb and suggest a significant depletion of the Eb peptide under knockdown conditions (Appendix A, Figure A3).

### 2.5. Effects of Eb Knockdown on Cell Proliferation and Viability, Rescue and Repair via Exogenous Eb Addition

To determine how Eb protein may influence cellular responses under UV stress conditions, proliferation and viability assays were performed in the three knockdown cell lines. Loss of Eb protein was associated with alterations in proliferation and viability in varying degrees, depending both on cell lines and the presence of stress. To assess whether the Eb protein could partially restore cellular responses, rescue and repair experiments were performed for each cell line under both basal and UV stress conditions.

**Figure 4:**
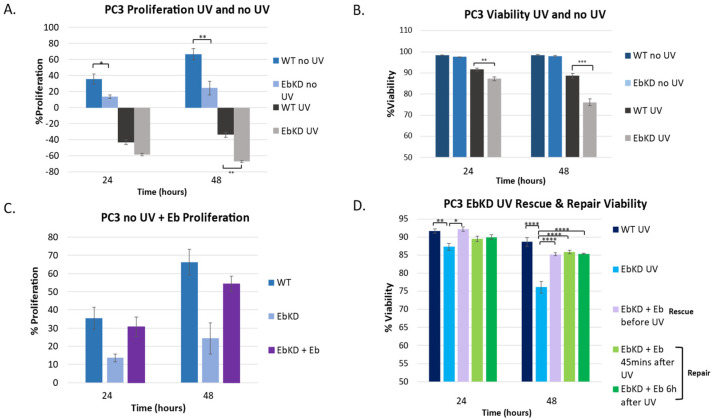
Proliferation and viability of PC3 WT, EbKD and EbKD rescue/repair groups under no UV and UV stress conditions, measured at 24 and 48 h. (**A**) Proliferation of WT and EbKD cells with and without UV exposure at 24 and 48 h. (**B**) Viability of WT and EbKD cells with and without UV exposure at 24 and 48 h. (**C**) Proliferation of WT, EbKD and EbKD + Eb without UV exposure at 24 and 48 h. (**D**) Viability of WT UV, EbKD UV and EbKD rescue (+Eb before UV) and repair groups (+Eb after UV) at 24 and 48 h. Bars represent mean ± SEM from three independent biological replicates. Asterisks indicate statistically significant differences based on Tukey’s HSD post hoc test (* *p* < 0.05, ** *p* ≤ 0.01, *** *p* ≤ 0.001, **** *p* ≤ 0.0001).

In the absence of UV exposure, PC3 EbKD cells exhibited reduced proliferation compared to WT at both 24 and 48 h (*p* < 0.01 at 48 h) (WT: 66.15% ± 7.05 vs. EbKD: 24.3% ± 8.6 at 48 h) (Figure 4A). Under UV treatment, the proliferation decreased in both groups compared to their no UV counterparts (*p* < 0.0001). Notably, PC3 EbKD cells maintained a much lower proliferation than WT cells under UV stress at both time points (*p* < 0.01 at 48 h, g = 6.4) (WT: −33.85% ± 3.1 vs. EbKD: −67.14% ± 1.43 at 48 h) (Figure 4A). As for the viability, under no UV exposure, it was similar in EbKD and WT cells (WT: 98.34% ± 0.06 vs. EbKD: 97.62% ± 0.06 at 24 h) (Figure 4B). Following UV stress, viability decreased in both groups and EbKD cells displayed a consistently lower viability than WT at both time points, with a significant difference observed at 48 h (*p* ≤ 0.0001 at 48 h, g = 4) (WT: 88.62% ± 1.2 vs. EbKD: 76.09% ± 1.6 at 48 h) (Figure 4B).

Exogenous administration of Eb protein to the EbKD cells, under no UV conditions, increased proliferation to levels comparable with WT cells (WT: 35.4% ± 6 vs. EbKD: 13.6% ± 2 vs. EbKD + Eb: 30.7% ± 5 at 48 h) (Figure 4C); however, the differences between EbKD and EbKD + Eb did not reach statistical significance (g = 2 at 48 h). Viability measurements under these conditions were comparable across the three groups (WT, EbKD and EbKD + Eb).

Rescue and repair experiments with exogenously administered Eb were also performed under UV stress for EbKD cells. Proliferation of EbKD UV rescue was slightly higher than that of EbKD UV at 24 h, while at 48 h, both EbKD rescue and EbKD + Eb (45 min after UV) had higher proliferation than EbKD UV, although the differences were not statistically significant (g = 2). In terms of viability, all three EbKD rescue and repair groups exhibited a significant increase compared to EbKD UV at 48 h (*p* < 0.0001, g = 4) (WT: 88.6% ± 1.21 vs. EbKD: 76.1% ± 1.6 vs. EbKD + Eb (before UV): 85.22% ± 0.39 vs. EbKD + Eb (45 min after UV): 85.88% ± 0.44 vs. EbKD + Eb (6 h after UV): 85.38% ± 0.14 at 48 h) (Figure 4D).

**Figure 5:**
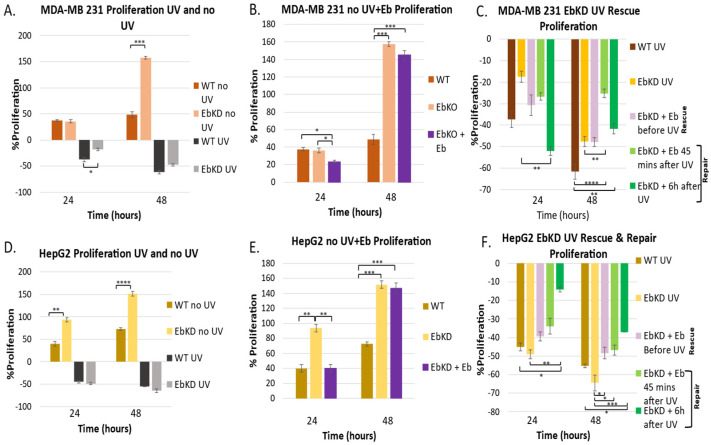
Proliferation of MDA-MB-231 and HepG2 WT, EbKD and EbKD rescue/repair groups under no UV and UV stress conditions, measured at 24 and 48 h. (**A**) Proliferation of MDA-MB-231 WT and EbKD cells with and without UV exposure at 24 and 48 h. (**B**) Proliferation of MDA-MB-231 WT, EbKD and EbKD + Eb without UV exposure at 24 and 48 h. (**C**) Proliferation of MDA-MB-231 WT UV, EbKD UV and EbKD rescue (+Eb before UV) and repair groups (+Eb after UV) at 24 and 48 h. (**D**) Proliferation of HepG2 WT and EbKD cells with and without UV exposure at 24 and 48 h. (**E**) Proliferation of HepG2 WT, EbKD and EbKD + Eb without UV exposure at 24 and 48 h. (**F**) Proliferation of HepG2 WT UV, EbKD UV and EbKD rescue (+Eb before UV) and repair groups (+Eb after UV) at 24 and 48 h. Bars represent mean ± SEM from three independent biological replicates. Asterisks indicate statistically significant differences based on Tukey’s HSD post hoc test (* *p* < 0.05, ** *p* ≤ 0.01, *** *p* ≤ 0.001, **** *p* ≤ 0.0001).

MDA-MB-231 cells displayed a different pattern of proliferation, with EbKD cells having much higher proliferation compared to WT control, at 48 h (*p* ≤ 0.0001, g = 1) (WT: 48.6% ± 5.7 vs. EbKD: 157.6% ± 2.6 at 48 h) (Figure 5A). Interestingly, after UV exposure, EbKD cells had better proliferation compared to WT UV, with the difference being statistically significant at 24 h but with a small effect size (*p* < 0.05, g = 0.3) (WT: −37.3% vs. EbKD: −17.5% ± 2.5 at 24 h) (Figure 5A). Viability was comparable between WT and EbKD, and although it fell slightly after UV stress for both groups, there were no significant differences between the viability of WT UV and EbKD UV.

Interestingly, MDA-MB-231 EbKD cells that received Eb exogenously without UV stress had slightly lower proliferation than both WT and EbKD at 24 h, with the differences being statistically significant (*p* < 0.05, g = 2) (WT: 37.4% ± 1.8 vs. EbKD: 35.9% ± 2.8 vs. EbKD + Eb: 23.3% ± 1.7 at 24 h) (Figure 5B). However, by 48 h the proliferation of both EbKD and EbKD + Eb had risen much higher than that of WT (*p* ≤ 0.0001) (WT: 48.6% ± 5.7 vs. EbKD: 157.6% ± 2.6 vs. EbKD + Eb: 145% ± 5 at 48 h) (Figure 5B). Nevertheless, there were no significant differences in the viability of these three groups.

**Figure A4:**
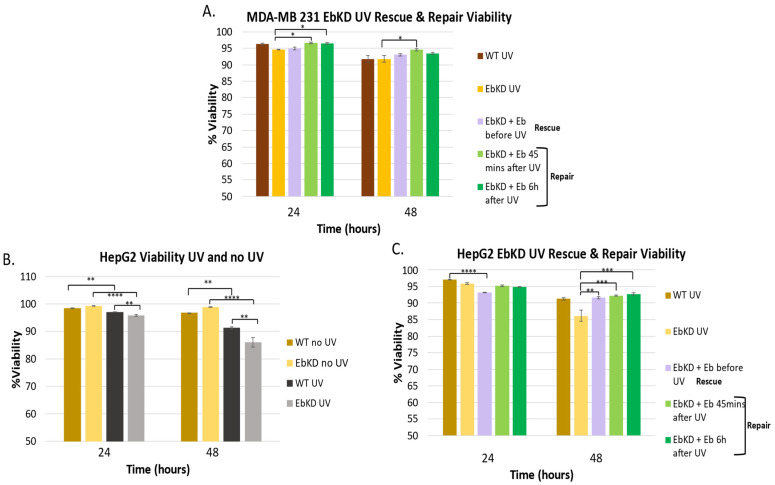
Proliferation and Viability of WT, EbKD, as well as EbKD rescue and repair groups for PC3, MDA-MB-231 and HepG2 cell lines, after UV radiation. (**A**) Viability of EbKD MDA-MB-231 rescue and repair groups did not show much difference compared to WT and EbKD UV controls. At 24 h the two repair groups had slightly higher viability than EbKD UV and at 48 h the early repair group (EbKD+Eb 45 min after UV) had a small increase in viability compared to EbKD UV. (**B**) Viability of HepG2 EbKD cells, was reduced after UV radiation compared to WT UV control. There were no significant differences in the viability of EbKD and WT under normal conditions. (**C**) Viability of all three HepG2 EbKD rescue and repair groups was significantly higher compared to EbKD UV at 48 h, at the same time there was no statistically significant difference between the rescue and repair groups and WT UV. Asterisks indicate statistically significant differences based on Tukey’s HSD post-hoc test (* *p* < 0.05, ** *p* ≤ 0.01, *** *p* < 0.001, **** *p* ≤ 0.0001).

Rescue and repair groups of EbKD exhibited some changes compared to the UV controls. Interestingly, at 24 h, EbKD + Eb (6 hours after UV) had significantly lower proliferation than every other EbKD + Eb group (*p* < 0.05), as well as EbKD UV (*p* < 0.01, g = 0.7). The difference with WT UV was also visible, but not statistically significant. At 48 h, repair group EbKD + Eb (45 min after UV) had the best proliferation compared to every other group, with the differences between it and EbKD UV, as well as WT UV being statistically significant (*p* < 0.01, g = 3.5) (WT UV: −61.7% ± 3.6 vs. EbKD UV: −47.7% ± 2.4 vs. EbKD + Eb (before UV): −47.9% ± 2.1 vs. EbKD + Eb (45 min after UV): −25.2% ± 1.9 vs. EbKD + Eb (6 h after UV): −41.7% ± 2.4 at 48 h) (Figure 5C). EbKD + Eb (45 min after UV) also exhibited a modest increase in viability compared to EbKD UV at both timepoints (*p* < 0.05, g = 1) (EbKD UV: 91.7% ± 1 vs. EbKD + Eb (45 min after UV): 94.5% ± 0.4 at 48 h) (Appendix A, Figure A4). However, the difference in mean values was minimal, so the statistical significance may reflect the low variance.

HepG2 cells followed a similar pattern of proliferation, with EbKD cells having noticeably higher proliferation compared to WT control, at both timepoints (*p* < 0.0001 at 48 h, g = 10) (WT: 72.5% ± 2.9 vs. EbKD: 151.6% ± 4.8) (Figure 5D). Under UV stress EbKD cells exhibited a slightly lower proliferation than WT UV control at both time points, although the difference was not proven to be statistically significant (Figure 5D). The viability of the two groups was similar without stress conditions, but following UV exposure, EbKD cells showed lower viability than WT (*p* < 0.05 at 48 h, g = 2) (WT UV: 91.3% ± 0.3 vs. EbKD: 86.1% ± 1.7) (Appendix A, Figure A4).

EbKD cells that received Eb exogenously exhibited lower levels of proliferation compared to EbKD (*p* < 0.01 at 24 h, g = 6) (WT: 40.1% ± 4.9 vs. EbKD: 93.5% ± 4.8 vs. EbKD + Eb: 40.3% ± 4.8 at 24 h), reaching levels similar to WT (Figure 5E). However, by 48 h, proliferation in the EbKD cells that were treated with Eb increased to levels comparable to the untreated knockdowns (Figure 5E). All EbKD UV rescue and repair groups exhibited a better outcome compared to EbKD UV. More specifically, at 24 h, EbKD + Eb (6 h after UV) had the best proliferation out of all the other groups, even compared to the WT UV control (*p* < 0.05) (WT UV: −45% ± 2.2 vs. EbKD UV: −49.2% ± 2.4 vs. EbKD + Eb (before UV): −39.5% ± 2.4 vs. EbKD + Eb (45 min after UV): −33.9% ± 4.3 vs. EbKD + Eb (6 h after UV): −14.2% ± 1.3 at 24 h) (Figure 5F). By 48 h, all rescue and repair groups had higher proliferation than EbKD UV control (*p* < 0.05, g = 2 to 4) and EbKD + Eb (6 h after UV) was significantly higher than WT UV as well (*p* < 0.05). This trend was also depicted in the viability, where all three rescue and repair groups showed higher viability, similar to that of WT UV, in comparison to EbKD UV control (*p* < 0.01 at 48 h, g = 2) (WT UV: 91.3% ± 0.32 vs. EbKD UV: 86.15% ± 1.7 vs. EbKD + Eb (before UV): 91.7% ± 0.3 vs. EbKD + Eb (45 min after UV): 92.3% ± 0.18 vs. EbKD + Eb (6 h after UV): 92.7% ± 0.3 at 48 h) (Appendix A, Figure A4).

### 2.6. Effects of EbKD on Ec Gene Expression

**Figure A5:**
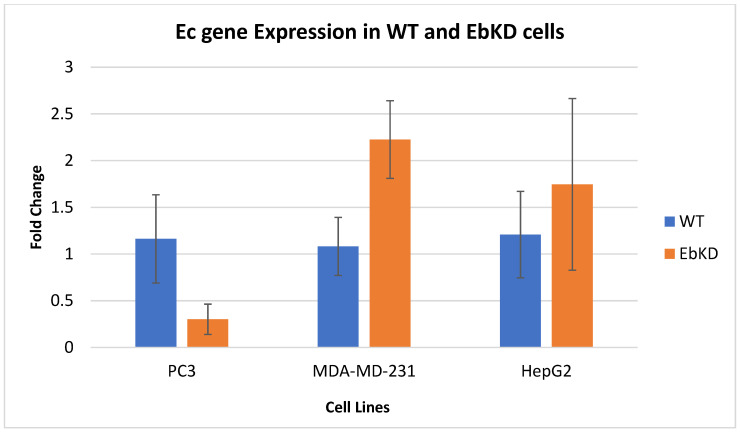
Relative expression of Ec gene in WT and EbKD PC3, MDA-MB-231 and HepG2 cells measured by quantitative real-time PCR. Ec expression was normalized to GAPDH and calculated using the 2^−ΔΔCt^ method from three independent biological replicates (n = 3). No statistically significant differences in Ec expression were observed between WT and EbKD cells in any of the cell lines analyzed. Data are presented as mean ± SEM.

To investigate whether Eb loss affects the expression of Ec peptide, the levels of Ec expression were examined through qRT-PCR for WT and EbKD PC3, MBD-MB231 and HepG2 cells. Ct values were normalized to the housekeeping gene GAPDH, and relative expression was calculated using the ΔCt method from three independent biological replicates (*n* = 3). No statistically significant differences in Ec expression were observed between WT and EbKD cells in any of the cell lines analyzed (Appendix A, Figure A5). In MDA-MB-231 cells, EbKD showed a trend towards increased Ec expression compared to WT (mean fold change 2.22 vs. 1.08), although variability between replicates was high and the difference did not reach statistical significance. In PC3 cells, Ec expression appeared reduced in EbKD cells relative to WT (0.30 vs. 1.16), again with substantial variability. In HepG2 cells, Ec expression levels were similar between WT and EbKD cells (1.74 vs. 1.21). Overall, the interpretation of these data is limited by inter-replicate variability and the exploratory nature of this analysis.

### 2.7. Apoptotic Cell Populations Identified by Flow Cytometric Annexin/PI Analysis

To evaluate how Eb loss and UV stress affect apoptotic responses, Annexin V/PI flow cytometric analysis was performed on WT and EbKD cells, with and without UV exposure. The percentages of viable, early apoptotic, late apoptotic and necrotic cells were then assessed.

**Figure 6:**
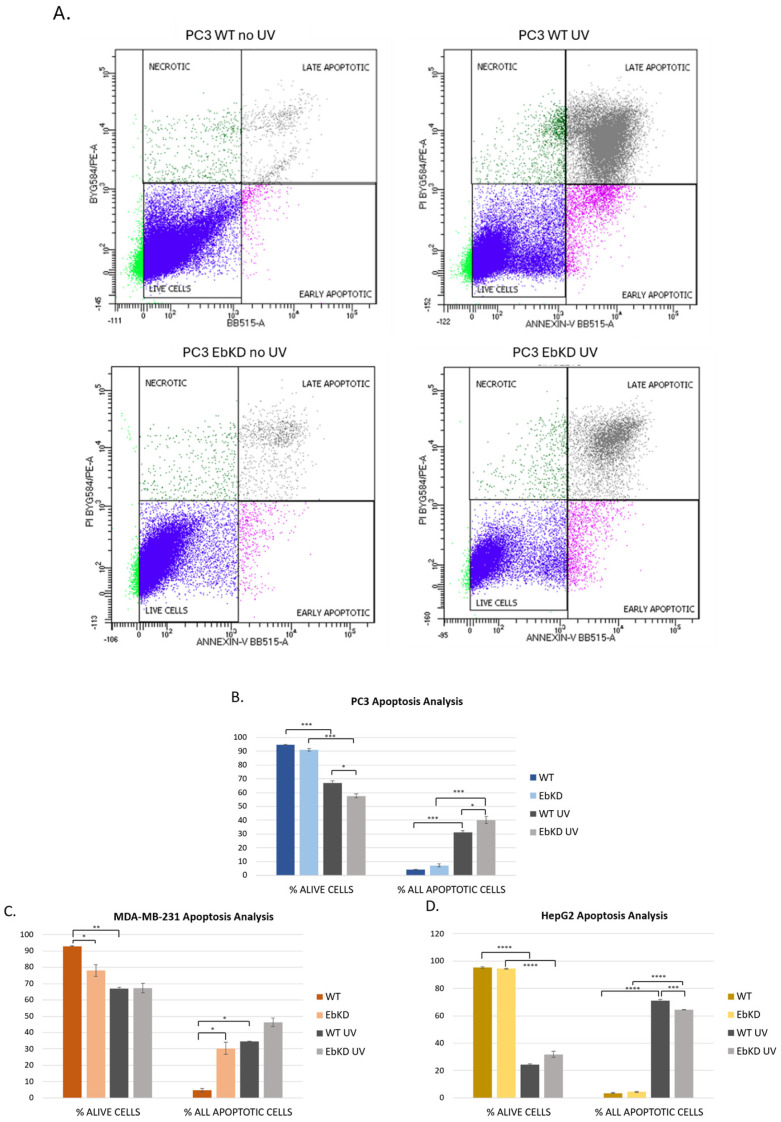
(**A**) Representative flow cytometry dot plots of Annexin V/PI analysis for PC3 WT and EbKD cells, with and without UV stress (blue: live cells, pink: early apoptotic cells, dark green: necrotic cells, grey: late apoptotic cells, light green: cells excluded by the gating threshold). (**B**) Percentages of alive and total apoptotic WT and EbKD PC3 cells, with and without UV stress. (**C**) Percentages of alive and total apoptotic WT and EbKD MDA-MB-231 cells, with and without UV stress. (**D**) Percentages of alive and total apoptotic WT and EbKD HepG2 cells, with and without UV stress. Asterisks indicate statistically significant differences based on Tukey’s HSD post hoc test (* *p* < 0.05, ** *p* ≤ 0.01, *** *p* < 0.001, **** *p* ≤ 0.0001).

In PC3 cells, no significant differences were observed between WT and EbKD under non-UV conditions. However, following UV exposure, EbKD had a higher total percentage of apoptotic cells (early + late apoptotic) than WT UV (*p* < 0.05) (WT UV: 31.3% ± 1.1 vs. EbKD UV: 40.15% ± 2.4), and subsequently the percentage of viable cells was lower in EBKD UV (*p* < 0.05) (WT UV: 66.9% ± 1.6 vs. EbKD UV: 57.7% ± 3.4) (Figure 6B). There were no significant differences in necrotic cells under either condition. MDA-MB-231 EbKD cells displayed elevated levels of total apoptosis compared to WT under no UV (*p* < 0.05) (WT: 4.7% ± 0.4 vs. EbKD: 30% ± 1.1) (Figure 6C). Under UV stress, however, the proportion of viable cells was comparable between WT and EBKD, and although EbKD UV cells showed a slightly higher total apoptotic percentage, the difference was not statistically significant.

In HepG2 cells, WT and EbKD showed similar apoptotic and viable percentages under no UV conditions (Figure 6D). After UV exposure, total apoptotic cells were higher in WT and EbKD compared to their no UV counterparts (*p* < 0.0001). WT after UV had a slightly higher percentage of total apoptotic cells in comparison with EbKD UV (*p* < 0.001) (WT UV: 71.15% ± 0.83 vs. EbKD UV: 64.4% ± 0.16) (Figure 6D).

### 2.8. Cell Cycle Phases Analysis in WT and EbKD Cells with and Without UV Stress

**Figure 7:**
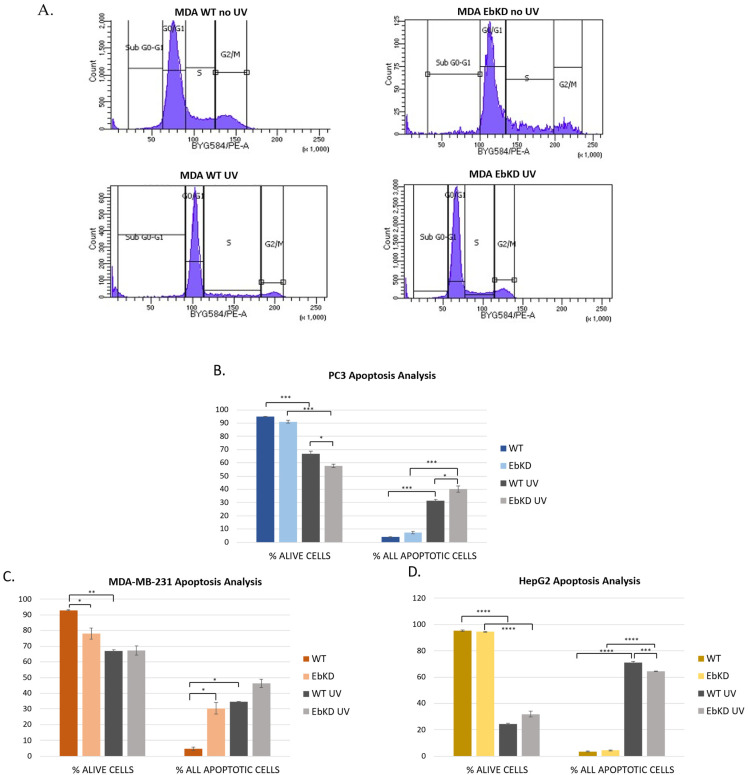
(**A**) Representative flow cytometry histograms of cell cycle profiles in MDA-MB-231 WT and EbKD cells, under normal conditions and under UV stress. (**B**) Quantification of cell cycle distribution in MDA-MB-231 WT and EbKD cells, with and without UV stress. (**C**) Quantification of cell cycle phase distribution in PC3 WT and EbKD cells, with and without UV stress. (**D**) Quantification of cell cycle phase distribution in HepG2 WT and EbKD cells, with and without UV stress. Asterisks indicate statistically significant differences based on Tukey’s HSD post hoc test (* *p* < 0.05, ** *p* ≤ 0.01, *** *p* < 0.001, **** *p* ≤ 0.0001).

To investigate whether Eb knockdown alters cell cycle, the distribution of cells in phases G1/G0, S, G2/M and sub-G0/G1 was analyzed using flow cytometry. In MDA-MB-231, the proportion of cells in G0/G1 was similar between WT and EbKD under both UV and no UV conditions. EbKD cells showed a significant reduction in S phase following UV stress, compared to EbKD no UV control (*p* < 0.01) (EbKD no UV: 21.1% ± 2.5 vs. EbKD UV: 10.8% ± 2.6), whereas S phase distribution in WT cells was not significantly affected by UV stress (Figure 7B). Additionally, EbKD cells also exhibited a lower G2/M phase compared to the WT control (*p* < 0.05) (WT no UV: 16.9% ± 1.03 vs. EbKD no UV: 10.95% ± 1.25) (Figure 7B), with no significant difference between the two groups under UV stress. Furthermore, the sub-G0/G1 population after UV stress was significantly lower in EbKD cells compared to WT (*p* < 0.01) (WT UV: 7.4% ± 0.3 vs. EbKD UV: 3.7% ± 0.3) (Figure 7B). PC3 cells were examined under the same conditions. The distribution of cells across the G0/G1, S and G2/M phases did not differ significantly between EbKD and WT, both under UV stress and no stress conditions. UV exposure significantly increased the sub-G0/G1 fraction in both EbKD and WT, compared to the no UV counterparts (*p* < 0.0001) (Figure 7C), consistent with elevated DNA fragmentation and cell death. However, no statistically significant difference was observed between WT and EbKD under UV stress (Figure 7C).

In HepG2, the G0/G1 phase was significantly reduced in EbKD cells after UV radiation compared to WT UV (*p* < 0.001) (WT UV: 52.2% ± 3.9 vs. EbKD UV: 19.55% ± 1.15) (Figure 7D). S phase distribution was comparable between all groups, while G2/M phase was significantly lower in UV-treated groups compared to the no UV controls (*p* < 0.01). However, no difference was observed between EbKD and WT within each condition. Sub-G0/G1 population was significantly elevated in EbKD cells compared to WT after UV radiation (*p* ≤ 0.0001) (WT UV: 22.6% ± 2.4 vs. EbKD UV: 38% ± 1.65) (Figure 7D).

### 2.9. Western Blot Analysis

**Figure 8:**
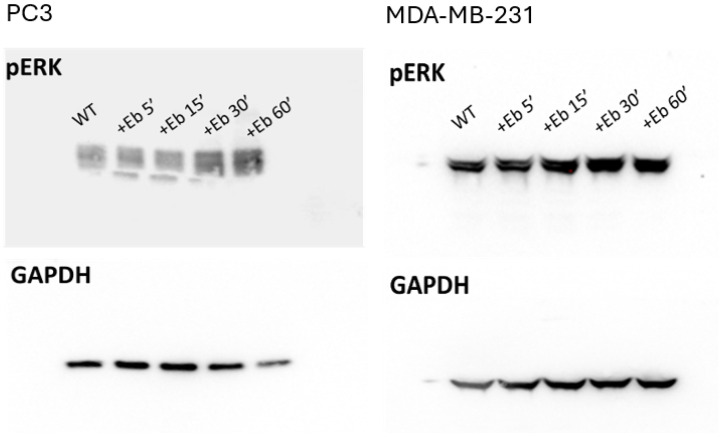
Exogenous Eb treatment and activation of signaling pathways. Time-course analysis of pERK1/2 activation following treatment with 50 nM of exogenous Eb in PC3 and MDA-MB-231 cells, compared with untreated controls. In PC3 cells, pERK1/2 levels were highest at 60 min after Eb treatment. In MDA-MB-231, only a modest increase in pERK was observed at 30–60 min. Protein levels were evaluated relative to GAPDH, which was used as a loading control.

To further examine the dynamics of ERK activation, another experiment was performed using 50 nM of exogenous Eb in PC3 and MDA-MB-231 cell lines. Total protein lysates were extracted at 5, 15, 30 and 60 min after treatment, and the levels of pERK were analyzed by Western blot (Figure 8). In PC3 cells, pERK levels were more pronounced at 60 min after Eb. In contrast, MDA-MB-231 cells exhibited only a modest increase in pERK levels at 30–60 min after Eb, with relatively small differences between the timepoints.

### 2.10. Effects of MAPK Inhibitor Addition on WT and EbKD Cell Proliferation and Viability

Proliferation and viability were assessed in PC3 WT and EbKD cells under the following treatment conditions: control, DMSO vehicle, inhibitor, Eb, and Eb + inhibitor, in the presence or absence of UV stress. DMSO alone did not significantly affect proliferation or viability compared to untreated controls.

**Figure 9:**
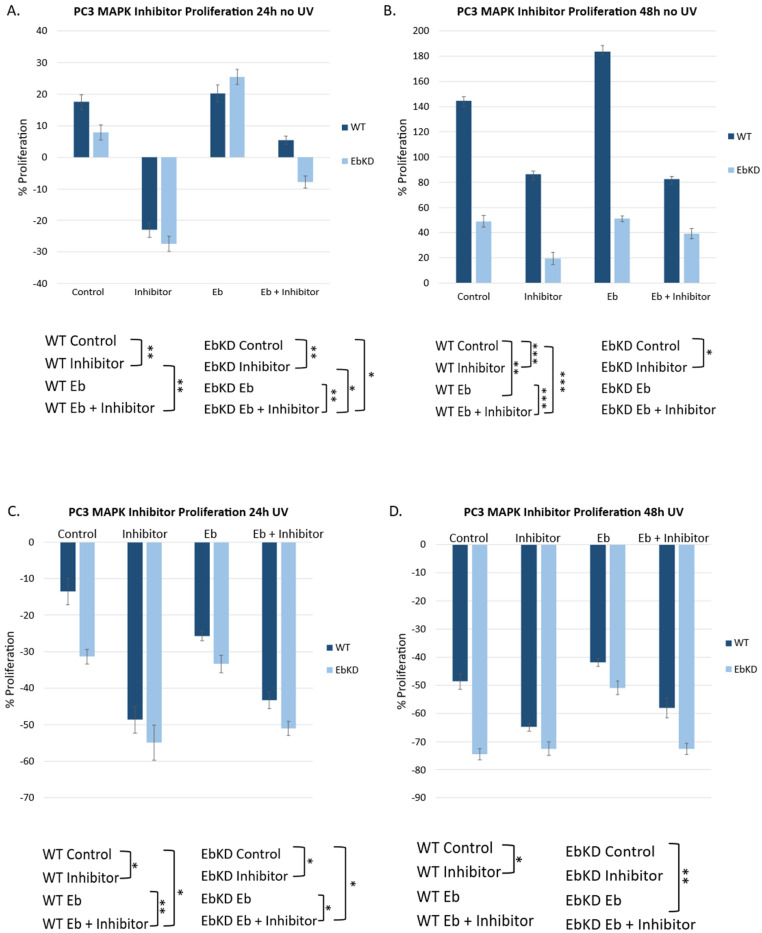
Effect of MAPK inhibitor on the proliferation of WT and EbKD PC3 cells under no-UV and UV stress conditions. (**A**,**B**). Proliferation percentages of WT and EbKD PC3 cells without UV stress at 24 h (**A**) and 48 h (**B**), treated with MAPK inhibitor, Eb peptide, and Eb + inhibitor. (**C**,**D**) Proliferation percentages of WT and EbKD PC3 cells after UV stress at 24 h (**C**) and 48 h (**D**), under the same treatment conditions. MAPK inhibition reduced proliferation in both WT and EbKD cells, with a more pronounced effect observed in WT cells, particularly at 48 h. Under UV stress, inhibitor-treated groups exhibited reduced proliferation compared to UV controls, while Eb treatment was associated with partial attenuation of UV-induced suppression, an effect that diminished upon co-treatment with the inhibitor. Bars represent mean ± SEM from three independent biological replicates. Asterisks indicate statistically significant differences based on unpaired t-tests with Bonferroni correction (* *p* < 0.05, ** *p* ≤ 0.01, *** *p* < 0.001).

Under non-UV conditions, the inhibitor significantly reduced proliferation in both WT and EbKD cells, with a more pronounced effect in WT cells (*p* < 0.001 at 48 h). At 48 h, the inhibitor and Eb + inhibitor groups exhibited comparable proliferation levels in both WT and EbKD cells. However, WT cells treated with Eb + inhibitor maintained significantly lower proliferation compared to WT controls (WT control: 144.6% ± 3.6 vs. WT + inhibitor: 86.5% ± 2.3 vs. WT + Eb + inhibitor: 82.4% ± 2.3; *p* < 0.001 at 48 h) (Figure 9B). In EbKD cells, the inhibitor also reduced proliferation at 48 h compared to control (*p* < 0.05), although no significant differences were observed among the remaining EbKD treatment groups at this time point (EbKD control: 49% ± 4.8 vs. EbKD + inhibitor: 19.6% ± 4.8) (Figure 9B).

Under UV stress, inhibitor-treated groups showed reduced proliferation compared to UV controls at 24 h in both WT and EbKD cells. This effect persisted at 48 h only in WT cells (*p* < 0.05) (WT UV control: −48.6% ± 2.7 vs. WT UV + inhibitor: −64.8% ± 1.3) (Figure 9D). Similarly, Eb + inhibitor treatment decreased proliferation relative to UV controls at 24 h (*p* < 0.05), but this difference was not sustained at 48 h. Notably, treatment with Eb alone resulted in higher proliferation compared to the Eb + inhibitor group in both WT and EbKD cells at 24 h (*p* < 0.01) (WT UV + inhibitor: −48.6% ± 3.6 vs. WT UV + Eb: −25.7% ± 1.4 vs. WT UV + Eb + inhibitor: −43.2% ± 2.3) (Figure 9C). Cell viability was not significantly affected by inhibitor treatment in either WT or EbKD cells, under both UV and non-UV conditions.

### 2.11. Proteome Analysis Results

Comparative proteomic analysis was performed in PC3 cells under four conditions: WT, EbKD, WT UV (6 h after UV) and EbKD UV (6 h after UV). Proteins showing statistically significant differences across groups were identified by ANOVA, and post hoc Tukey testing was used to generate pairwise comparisons. Proteins were further filtered using pathway-related keywords associated with DNA damage response (DDR), base excision repair (BER), and nucleotide excision repair (NER). In total, 267 proteins were identified in the DDR/DNA repair cluster, 19 proteins in BER, and 14 proteins in NER.

**Figure 10:**
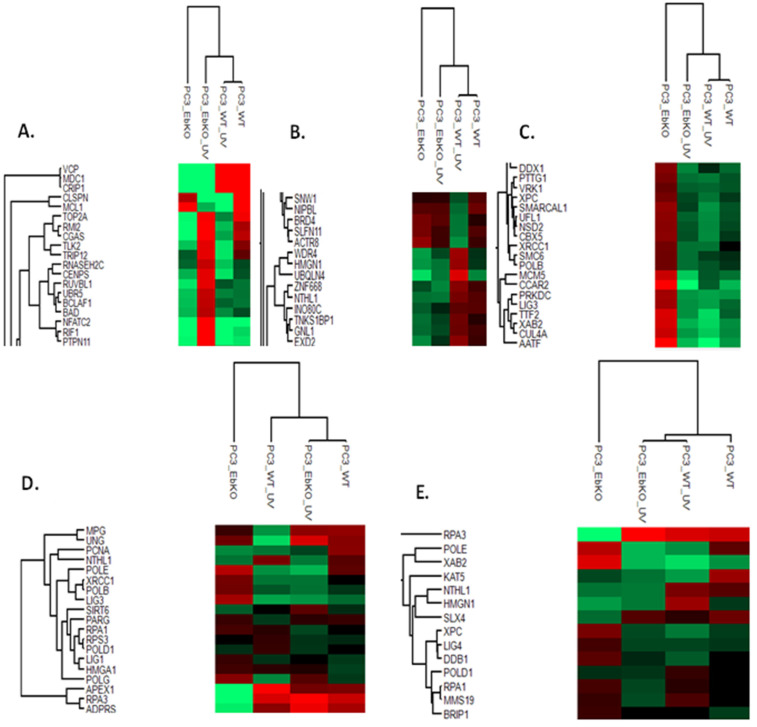
Proteomics comparison of PC3 WT, EbKD, WT UV and EbKD UV cells. (**A**–**C**) Heatmap of proteins associated with the DNA damage response (DDR) and DNA repair. Due to the large number of proteins, selected sections of the heatmap are presented to highlight some prominent clusters. (**D**) Heatmap of proteins associated with the base excision repair (BER) pathway. (**E**) Heatmap of proteins associated with the nucleotide excision repair (NER) pathway.

For the DDR proteins, MDC1 showed higher abundance in WT and WT UV cells, but was downregulated in EbKD and EbKD UV. CLSPN was upregulated in WT and EbKD cells, while it was downregulated in both of the following UV treatments: PARP1 was downregulated in WT and EbKD UV, while it was upregulated in WT UV and EbKD. BRD4 and SLFN11 were both downregulated only in WT + UV cells, while their expression in other groups was upregulated (Figure 10A–C).

Within the BER pathway, XRCC1 and LIG3 were upregulated in EbKD cells, while NTHL1 was upregulated in WT and WT UV cells but downregulated in EbKD and EbKD UV cells (Figure 10D). In the NER pathway, XPC and DDB1 were upregulated in EbKD cells, whereas RPA1 was downregulated in EbKD UV, and POLD1 was downregulated in both EbKD and EbKD UV cells (Figure 10E).

## 3. Discussion

The present study explores the potential role of the IGF-1Eb in cancer cell adaptation to stress, highlighting its context-dependent effects on proliferation and survival under UV-induced stress. By combining exogenous Eb peptide supplementation, targeted silencing, viability assays, wound healing analysis, flow cytometry, signaling analysis, and comparative proteomic profiling, we show that IGF-1Eb is associated with context-dependent effects across tumor types, with observations that suggest a possible contribution to cellular recovery following damage.

The transient nuclear entry of Eb peptide after UV exposure is consistent with the possibility that this isoform participates in early stress adaptation rather than sustained nuclear signaling. Its brief accumulation in the nucleus at 1 h, followed by enhanced cytoplasmic presence at 2 h, may indicate a rapid and temporally restricted association with early damage-response pathways before returning to cytoplasmic processes associated with recovery. Notably, the persistent nuclear retention of Eb under DRB-mediated transcriptional inhibition indicates that its initial nuclear import is unlikely to depend on active transcription but may instead be associated with upstream stress-sensing or chromatin-associated processes.

Across the models examined, exogenous Eb supplementation exerted moderate and context-dependent effects rather than acting as a uniformly pro-proliferative factor. Under basal conditions, Eb produced only modest increases in proliferation, indicating that while it may support normal growth, it does not function as a strong driver of cell expansion. Under UV-induced stress, however, its influence became more evident and strongly shaped by both cell identity and the timing of administration.

In prostate (PC3, DU145) and BC cell lines (MCF-7, MDA-MB-231), Eb pretreatment generally improved recovery at later time points, with significant increases in proliferation at 48 h in DU145 and MCF-7. These observations suggest that early exposure may partially support cellular adaptation to the forthcoming genotoxic insult. PC3 cells, although initially unresponsive, showed clearer recovery when Eb was administered shortly after UV irradiation, indicating that the peptide may further contribute to processes associated with post-damage recovery rather than prevention alone. In contrast, liver-derived lines (Huh-7, HepG2) exhibited milder responses, with only modest improvements regardless of timing, highlighting a lower sensitivity to the peptide in this cellular context.

The combined findings from the proliferation, viability, migration, rescue, and cell-cycle experiments across PC3, MDA-MB-231, and HepG2 cell lines collectively point toward a context-dependent role for the Eb peptide in coordinating cellular responses to stress rather than uniformly promoting proliferation. Immunofluorescence confirmed efficient peptide depletion in all EbKD cell lines, supporting the interpretation that the observed phenotypic changes are associated with reduced Eb expression.

Under non-stressed conditions, PC3 demonstrated the clearest dependence on endogenous Eb. Silencing the peptide led to a marked reduction in proliferation at both 24 and 48 h, and although wound closure was not statistically altered, the trend toward slower migration may indicate that Eb contributes to sustaining proliferative output and cellular motility in this prostate cancer model. HepG2 cells showed an almost opposite pattern: proliferation increased significantly after Eb depletion, indicating that in hepatocellular carcinoma cells, Eb may function as a growth-modulating factor rather than a proliferative driver.

Migration capacity, however, was modestly reduced, implying that Eb may support cytoskeletal or adhesion-related processes independently of its effect on proliferation. In contrast, MDA-MB-231 cells exhibited only minimal differences in growth without stress and no detectable migration defects, which may reflect the extensive signaling redundancy characteristic of TNBC cells. Collectively, these observations highlight that the biological role of Eb is strongly shaped by lineage-specific signaling environments.

A more consistent pattern emerged under UV stress. All WT cells responded to UV exposure with reduced proliferation and viability, as expected. However, Eb depletion generally exacerbated these effects, suggesting a potential protective role for the peptide during genotoxic stress. PC3 EbKD cells were the most strongly affected, with markedly reduced proliferation and viability compared with WT controls, indicating impaired stress adaptation.

Rescue experiments further supported the functional importance of EB. In PC3 cells, exogenous peptide supplementation improved proliferation under both basal and UV conditions, with the most pronounced effect observed when Eb was added shortly after UV exposure. Viability increased significantly across rescue conditions, indicating that Eb supplementation may partially restore processes associated with stress recovery.

Flow cytometric analysis of apoptosis and cell-cycle distribution revealed that loss of the Eb was associated with distinct stress-response patterns across cell lines. Under non-UV conditions, PC3 and HepG2 cells showed no major shifts in viability or apoptotic burden following EbKD, indicating that the peptide is not required for maintaining homeostatic survival in these lines. By contrast, MDA-MB-231 cells exhibited a substantial increase in apoptosis in the absence of UV when Eb was depleted, suggesting that this highly aggressive TNBC line may rely on the peptide to help maintain baseline apoptotic resistance.

Upon UV exposure, however, the functional role of Eb became more evident across all models. PC3 EbKD significantly heightened apoptotic susceptibility, with greater total apoptosis and reduced viability compared with WT UV. This increased sensitivity was mirrored in the cell-cycle profile, where both WT and EbKD cells showed the expected rise in sub-G0/G1 after UV exposure, consistent with DNA fragmentation, but without a significant difference between genotypes, indicating that PC3 cells, more sensitive to Eb depletion under stress conditions, respond to UV through shared terminal pathways regardless of peptide status. MDA-MB-231, in contrast, showed a blunted UV-induced phenotype: viability after UV was comparable between WT and EbKD cells, and although EbKD UV exhibited slightly higher apoptosis, the difference was not significant.

Additionally, their cell-cycle distribution largely remained intact, apart from an EbKD-specific drop in S-phase after UV, suggesting that while the peptide contributes to replicative stress management, TNBC cells may retain compensatory signaling responses that limit apoptotic sensitivity. HepG2 cells followed yet another pattern. Under normal conditions, EbKD and WT displayed nearly identical apoptotic and cell-cycle profiles, but after UV exposure, the alterations associated with Eb depletion in cell-cycle progression became more evident. G0/G1 fraction dropped sharply in EbKD UV cells, while the sub-G0/G1 population was markedly elevated compared to WT, indicating extensive DNA fragmentation and impaired checkpoint retention. Surprisingly, WT HepG2 displayed slightly higher total apoptosis than EbKD under UV, suggesting that although loss of Eb disrupts cell-cycle control, it does not necessarily exacerbate Annexin measured apoptotic death in this context, possibly because damaged EbKD HepG2 are diverted away from classical apoptotic pathways toward rapid fragmentation or necrotic-like outcomes.

Western blot analysis was used to explore whether Eb exposure influences major intracellular signaling pathways. To investigate the temporal dynamics of this response, p-ERK was examined in a time-course experiment following treatment with 50 nM Eb. In PC3 cells, pERK levels gradually increased and appeared most pronounced at approximately 1 h after stimulation. MDA-MB-231 cells, however, showed a more limited response, with only a modest elevation in p-ERK1/2 detected at later time points and relatively small differences between the intervals examined. This reduced responsiveness may be related to the constitutive activation of MAPK signaling frequently associated with KRAS pathway alterations in these cells, which might diminish the magnitude of ERK activation in response to external stimuli. Although these findings do not establish a direct mechanistic link, they suggest that Eb exposure may influence ERK-associated signaling responses in a cell-type-dependent manner.

MAPK inhibition highlights possible involvement of this pathway in mediating Eb-associated effects. The pronounced suppression of proliferation following inhibitor treatment, particularly in WT cells, together with the inability of exogenous Eb to counteract this reduction, suggests that MAPK activity may contribute to Eb-driven responses. Under UV stress, inhibitor treatment further limited proliferative recovery, effectively blunting the supportive effect of Eb, supporting a role for MAPK signaling in stress adaptation. These findings are consistent with the possibility that Eb may act upstream of, or in convergence with, MAPK-dependent proliferative signaling during the early phases of recovery following damage. In contrast, cell viability remained largely unchanged, indicating that the impact of MAPK inhibition is primarily restricted to proliferative capacity rather than survival.

Proteomic analysis provided additional insight into the potential impact of Eb depletion on cellular responses to DNA damage. Comparative proteomic profiling of PC3 cells under four experimental conditions (WT, EbKD, WT UV, and EbKD UV) identified multiple proteins with differential abundance across groups. Filtering of the dataset using DDR-related keywords revealed substantial changes in the abundance of DDR-associated proteins, along with smaller subsets of differentially expressed proteins linked to BER and NER pathways.

Within the DDR-associated proteins, several regulators displayed condition-dependent expression patterns. MDC1 showed higher abundance in WT and WT + UV cells but was reduced in Eb-depleted conditions, suggesting that Eb loss may influence components involved in early DNA damage signaling. CLSPN was elevated in both WT and EbKD cells but decreased following UV exposure in both backgrounds, while PARP1 displayed the opposite trend, showing higher levels in WT UV and EbKD cells, but lower abundance in WT and EbKD UV conditions. In addition, BRD4 and SLFN11 were selectively reduced in WT cells following UV exposure, whereas their expression remained relatively higher in the other conditions. Together, these patterns suggest that Eb depletion may influence proteins associated with checkpoint signaling, chromatin-associated stress responses, and replication-related processes.

Moreover, alterations were observed in proteins associated with specific DNA repair pathways. Within the BER-related proteins, XRCC1 and LIG3 were elevated in EbKD cells, whereas NTHL1 showed higher expression in WT and WT UV cells but decreased levels following Eb depletion. Similarly, several NER-associated proteins displayed differential abundance across experimental conditions. XPC and DDB1 were increased in EbKD, whereas RPA1 and POLD1 showed reduced levels following Eb depletion, particularly after UV exposure. Because BER and NER pathways are central to the repair of oxidative DNA damage and UV-induced lesions, these changes may reflect alterations in proteins associated with DNA damage response pathways following Eb depletion.

Overall, the findings presented here indicate that IGF-1Eb does not appear to function as a universal proliferative factor but rather appears to modulate cellular responses in a context-dependent manner. While its influence under basal conditions varies between tumor cell lines, a more consistent pattern emerges following UV-induced stress, with Eb depletion leading to impaired cellular recovery. Together with the signaling and proteomic observations, these results suggest that IGF-1Eb may be associated with broader cellular stress-response networks. Further studies will be required to define the molecular pathways through which this isoform influences damage-associated signaling and cellular adaptation.

## 4. Materials and Methods

### 4.1. Cell Lines

Six human cancer cell lines were employed in this study: MDA-MB-231 (triple-negative breast adenocarcinoma) (CN: ATCC-HTB-26, ATCC, Bethesda, MD, USA) and MCF-7 (ER-positive breast adenocarcinoma) (CN: ATCC-HTB-22, ATCC, Bethesda, MD, USA), PC-3 (androgen-independent, p53-null prostate adenocarcinoma) (CN: ATCC-CRL-1435, ATCC, Bethesda, MD, USA) and DU145 (androgen-independent prostate carcinoma) (CN: ATCC-HTB-81, ATCC, Bethesda, MD, USA), and HepG2 (well-differentiated hepatocellular carcinoma) (CN: ATCC-HB-8065, ATCC, Bethesda, MD, USA) and Huh-7 (moderately differentiated hepatoma) (CΝ: 300156, Cytion, Heidelberg, Germany). All cell lines were purchased from the American Type Culture Collection (ATCC, Bethesda, MD, USA). MDA-MB-231, PC-3, and HepG2 cells were cultured in Dulbecco’s Modified Eagle Medium (DMEM; Capricorn Scientific, Ebsdorfergrund, Germany), supplemented with 10% heat-inactivated fetal bovine serum (FBS; Biochrom, Berlin, Germany), and maintained at 37 °C in a humidified atmosphere containing 5% CO_2_ (Forma Scientific, Marietta, OH, USA). Medium was replenished every 2 days, and cells were sub-cultured at 70–80% confluence using 0.25% trypsin-EDTA (Gibco, Thermo Fisher Scientific, Waltham, MA, USA). Cell cultures were regularly inspected microscopically for changes in morphology or growth characteristics that could indicate contamination; no evidence of contamination was observed during the study.

### 4.2. Electrophoretic Mobility Shift Assay (EMSA)

Electrophoretic mobility shift assays were performed to assess the ability of IGF-1Eb to interact with DNA. A 1% (*w*/*v*) agarose gel was prepared in 1× TBE buffer supplemented with ethidium bromide (2.5 µL/100 mL). Recombinant IGF-1Eb or IGF-1Ea peptides were incubated with the DNA probe under binding conditions for 20–30 min at room temperature. Where indicated, a rabbit polyclonal anti-human-IGF-1Eb antibody (in-house generated) was added to the reaction mixture to confirm binding specificity through supershift formation. Following incubation, samples were mixed with loading dye and loaded onto the agarose gel. Electrophoresis was conducted at 100 V until clear separation of free DNA and DNA–protein complexes was achieved. Bands were visualized under UV illumination using a transilluminator. A shift in DNA mobility relative to the DNA-only control was interpreted as DNA–protein complex formation, whereas an additional retardation in the presence of the antibody indicated a supershift and verified isoform-specific DNA binding by IGF-1Eb.

### 4.3. Exogenous Eb Peptide Treatment

The optimal working concentration of the Eb peptide (>95% purity, SciLight Biotechnology, Beijing, China) was first determined. A range of Eb peptide concentrations was tested, and cell responses were subsequently evaluated to identify the most effective dose. Based on these preliminary dose–response experiments, the concentration of 25 nM was selected as the optimal condition. This concentration was then used in all subsequent proliferation and migration assays.

### 4.4. DRB (5,6-dichloro-1-β-dribofuranosylbenzimidazole)

PC3 cancer cells were maintained in DMEM supplemented with 10% FBS at 37 °C in a humidified 5% CO_2_ incubator. The transcription inhibitor DRB (Sigma-Aldrich, St. Louis, MO, USA) was prepared as a 100 mM stock solution in PBS and added to the culture medium at a final concentration of approximately 100 µM for 1 h prior to UV exposure. Following DRB treatment, cells were washed with PBS and irradiated in a UV crosslinker (UVITEC Cambridge CL-508 Crosslinker, Cambridge, UK) under standardized conditions at a wavelength of 254 nm (50/60 H, 230 V, 65 W) with a dose of 30 J for 30 s. Samples were placed at the center of the UV crosslinker chamber at a fixed distance from the UV source, as indicated by the manufacturer’s positioning mark. Three experimental groups were analyzed: UV + PC3 control, UV + DRB, in which cells were irradiated without the UV filter, and UV-only, in which irradiation was performed using the UV filter and without DRB treatment. Control plates were handled in parallel but were not exposed to UV. After irradiation, cells were either collected immediately or returned to the incubator for the designated recovery intervals. The same samples were monitored across all time points to ensure consistency and comparability across experimental conditions.

### 4.5. Plasmid Preparation and Extraction

IGF-1Eb was silenced in wild-type (WT) cell lines mentioned above through stable siRNA production using a plasmid created with psiRNA-h7SKhygro G1 Kit (Sigma-Aldrich, St. Louis, MO, USA) according to the manufacturer’s instructions. Complementary oligonucleotides compatible with BbsI restriction enzyme were designed using the siRNA Wizard software [M56.1] (siRNAWizard™, Seattle, WA, USA; https://sirnawizard.com) and were inserted into the psiRNA-h7SKhygro vector following the manufacturer’s protocol. The oligonucleotides used were: ACCTCGGAGGGAGATTGGAAGTAGAATCAAGAGTTCTACTTCCAATCTCCCTCCTT (forward) and CAAAAAGGAGGGAGATTGGAAGTAGAACTCTTGATTCTACTTCCAATCTCCCTCCG (reverse). Confirmation of the siRNA insertion was performed through SpeI enzyme digestion and bidirectional sequencing using the primers provided by the kit (forward OL559 and reverse OL408). The ligated plasmid was transformed into *Escherichia coli* (GT116 strain) via heat shock, and transformed bacteria were selected using blue/white screening on XGal-containing agar.

Plasmid DNA was then extracted from overnight bacterial cultures, using QIAprep Spin Miniprep Kit (QIAGEN, Hilden, Germany), according to the manufacturer’s instructions. The concentration and purity of the extracted plasmid DNA were assessed using the Cytation5 Imaging Reader (BioTek Instruments, Winooski, VT, USA). Absorbance was measured at 260, 280 and 230 nm, and RNA purity was evaluated using the A260/A280 and A260/A230 ratios, and only plasmid samples with A260/A280 values between 1.8 and 2.0 were used for transfection.

### 4.6. Transfection

Before transfection, a hygromycin cytotoxicity assay was performed to determine the optimal concentration of antibiotic for selecting the transfected cells. Cells were seeded on a 24-well plate until ~80% confluent. Cell counts were taken before treatment, then cells were treated with varying hygromycin concentrations. Counts were repeated at 48 and 72 h. The concentration causing 50% cell death for each cell line was chosen for the selection. More specifically, 50 μg/mL was used for MDA-MB-231, 150 μg/mL for HepG2 and 50 μg/mL for PC3.

Following the confirmation of the optimal selection conditions, cells were transfected using TurboFect Transfection Reagent (Thermo Fisher Scientific, Waltham, MA, USA). Cells were seeded in 24-well plates at 60–70% confluency in 1 mL of DMEM. Plasmid DNA (0.7–1 μg per well) was diluted with serum-free OPTIMEM to a final volume of 100 μL per well. Turbofect transfection reagent was briefly vortexed, and 1 μL of Turbofect per well was added directly to the diluted DNA solution. The DNA-reagent mixture was gently mixed by pipetting and incubated at room temperature for 30 min. This transfection mixture was then added dropwise to the cells, without removing the growth medium from the wells. The cells were then incubated at 37 °C for 5 h; then, the medium was replaced with fresh growth medium, after gently washing the cells once with PBS. Twenty-four hours post-transfection, antibiotic selection was initiated using the optimized hygromycin concentrations and maintained for 6–7 days, with the medium refreshed every other day.

### 4.7. Immunofluorescence Staining

Immunofluorescence staining was carried out to observe the Eb peptide’s localization after UV treatment and to confirm the knockdown of the Eb protein. Cells were seeded onto 8-well chamber slides and cultured until at least 70–80% confluence. The cells were washed twice with PBS and fixed with 4% paraformaldehyde (PFA) for 30 min at room temperature. Following washes with cold PBS, cells were incubated with a custom-made rabbit anti-Eb primary antibody (1:100 in PBS) for 2 h at room temperature. After washing, cells were incubated with Alexa Fluor 488-labeled anti-rabbit secondary antibody (1:100), with DAPI (Life Technologies, Carlsbad, CA, USA) (1:1000) added at 10% of the secondary antibody volume, for 1 h in the dark. Cells were washed again, and the coverslips were left to dry in the dark and stored at 4 °C until imaging. Images were taken using the EVOS M7000 Imaging System (Thermo Fisher Scientific, Waltham, MA, USA).

### 4.8. Proliferation and Viability Assays

For each cell line, WT and EbKD cells were seeded in triplicate wells in 24-well plates under the following conditions: WT with UV stress, EbKD with UV stress, WT without UV stress and EbKD without UV stress. After allowing cells to adhere to the wells for 24 h, the growth medium (DMEM) was removed, and the UV groups were exposed to 30 J of UV radiation for 30 s. This was marked as time zero. Adherent cells were trypsinized at 0, 24 and 48 h, and counted using a hemocytometer. The culture medium containing non-adherent (dead) cells was also collected and centrifuged at 2800 rmp for 10 min. The pellet was resuspended in a 1:1 mixture of PBS and Trypan blue, and the dead cells were counted using a Neubauer hemocytometer (Marienfeld, Lauda-Königshofen, Germany). The number of alive and dead cells was used to calculate the percentage of proliferation and the percentage of viability for each condition. Proliferation was calculated as the relative increase in cell number at 24 and 48 h compared to the initial cell number at 0 h [% Proliferation = ((cell number t_x_ − cell number t_0_)/cell number t_0_) × 100]. Cell viability was calculated as the percentage of live cells relative to the total number of cells [% Viability = (live cells/total cells) × 100].

### 4.9. Rescue and Repair Assays

For the exogenous Eb rescue and repair experiments, proliferation and viability were assessed in four WT conditions: untreated WT, WT exposed to UV, WT pre-treated with Eb 45 min before UV, and WT receiving Eb 45 min after UV exposure. For the rescue conditions, proliferation and viability assays were performed as described above, using the same timepoints (0, 24, and 48 h). For EbKD cell lines, the experimental groups included WT and EbKD cells with and without UV stress. There were additional groups with exogenously administered Eb protein (25 nM), which included EbKD cells with exogenous Eb without UV and UV-stressed EbKD cells with exogenous Eb added at three different timepoints—45 min before UV exposure and 45 min and 6 h after UV exposure.

### 4.10. Wound Healing Assay

Cell migration was evaluated using a wound healing assay performed under four experimental conditions: untreated WT, WT + UV, WT + Eb, and WT + UV + Eb. For the Eb-supplemented groups, recombinant Eb peptide was added immediately after scratching at the optimized concentration of 25 nM, while UV-treated groups were irradiated with UV immediately before medium replacement. In parallel, for the EbKD migration experiments, WT and EbKD derivatives of each cell line were processed, allowing direct comparison of migration capacity under endogenous Eb depletion. The cells were seeded in 24-well plates and grown to a confluency of 80–90%, forming a monolayer. After removing the growth medium, a scratch was created using a 200 μL pipette tip. Following the scratch, the wells were gently washed with PBS, and 1 ml of fresh growth medium (DMEM) was added to each well. Wound closure was monitored using a Nikon Eclipse TS100 inverted phase-contrast microscope equipped with a digital camera, at 4× magnification. Images were captured immediately after scratch (time 0) and every 6 h up to 24 h. Each condition (cell line x time point) was assessed in triplicate wells, and images from each well and time point were analyzed using ImageJ software v0.6.0 (https://imagej.net). At least three distances across the wound area were measured per image to assess the width, and the percentage of wound closure was calculated accordingly using Microsoft Excel [% Wound closure = ((W_0_ − W_t_)/W_t_) × 100]. To minimize bias, image analysis was conducted without referencing the previous measurements.

### 4.11. Quantitative Real-Time PCR (qRT-PCR)

Total RNA was isolated from the three cell lines (WT and EbKD) (*n* = 3 biological replicates, each measured in technical triplicate) using TRIzol Reagent (Invitrogen, Carlsbad, CA, USA) according to the manufacturer’s instructions. Reverse transcription was carried out using the PrimeScript™ RT Reagent Kit (Takara Bio Inc., Shiga, Japan). cDNA synthesis was performed using the Veriti™ 96-Well Thermal Cycler (Applied Biosystems, Waltham, MA, USA). Quantitative real-time PCR was carried out using the QuantStudio™ 5 Real-Time PCR System (Applied Biosystems, Thermo Fisher Scientific, Waltham, MA, USA) with KAPA SYBR FAST Bio-Rad iCycler (Kapa Biosystems, Wilmington, MA, USA). Each 10 μL reaction contained 2 μL of cDNA, SYBR Green master mix, and gene-specific primers for IGF1-Ec, diluted 1:10 from stock (diluted in nuclease-free water). Primer sequences are listed in Table 1. All reactions were performed in triplicate.

The PCR conditions were as follows: initial denaturation at 95 °C for 2 min, followed by 45 cycles of 95 °C for 10 s and 60 °C for 25 s. A melt curve analysis was included with steps at 95 °C 10 s, 60 °C 20 s, and 95 °C 15 s, to confirm amplification specificity. Expression levels were normalized to the housekeeping gene GAPDH, and relative quantification was performed using the 2^−ΔΔCt^ method.

### 4.12. Apoptosis Detection

BD Pharmingen FITC Annexin V Apoptosis Detection Kit I (BD Biosciences, Franklin Lakes, NJ, USA) was used to study the effect of Eb knockdown on apoptosis. The groups that were examined were WT and EbKD cells with and without UV stress. Cells were seeded two days prior to Annexin staining, and the UV-stress groups were exposed to UV radiation 24 h before staining. All the samples were prepared according to the manufacturer’s instructions. Trypsinized adherent cells and detached cells in the culture media were collected, combined and washed with cold PBS. The pellets were resuspended in Binding Buffer, and 100 μL of each sample was used for staining with Annexin V-FITC and propidium iodide (PI). The staining was performed, as per protocol, for 15 min in the dark, and finally, 400 μL of Binding Buffer (1×) was added to each tube. The samples were then analyzed using BD FACSymphony A3 Cell Analyzer (BD Biosciences, Franklin Lakes, NJ, USA). Cells were gated to exclude debris and doublets. The populations of viable, early apoptotic, late apoptotic and necrotic cells were quantified relative to singlets. The percentages were then normalized relative to the sum of the analyzed singlets.

### 4.13. Cell Cycle Analysis

Cell cycle distribution was assessed for WT and EbKD cells with and without UV stress. The cells were seeded two days prior to cell cycle analysis, and UV groups were exposed to radiation 24 h before analysis. After harvesting, cells were washed twice with PBS and fixed by adding 75% cold ethanol dropwise while gently vortexing to prevent clumping. Samples were stored at 4 °C for at least 1 h, washed twice to remove residual ethanol, then stained with PI/RNase Staining Buffer (BD Pharmingen; BD Biosciences, Franklin Lakes, NJ, USA) for 15 min at room temperature in the dark and finally analyzed using BD FACSymphony A3 Cell Analyzer (BD Biosciences, Franklin Lakes, NJ, USA).

### 4.14. Western Blot

PC3 and MDA-MB-231 cells were treated with exogenous Eb protein. In a separate time-course experiment, PC3 and MDA-MB-231 cells were treated with 50 nM Eb, and protein lysates were collected at 5, 15, 30, and 60 min following treatment. Cells were lysed using RIPA buffer (50 mmol/L Tris-HCl, 150 mmol/L NaCl; Sigma-Aldrich) containing 0.5% Nonidet P-40, protease inhibitors (1 mmol/L phenylmethylsulfonyl fluoride (PMSF), 10 μg/mL aprotinin, and 10 μg/mL leupeptin; Sigma-Aldrich), and phosphatase inhibitors (1 mmol/L sodium orthovanadate and 1 mmol/L NaF; Sigma-Aldrich). Lysates were incubated on ice for 30 min and centrifuged at 19,722 *g* for 15 min at 4 °C. Protein concentrations were determined using the Bio-Rad Protein Assay (Bio-Rad Laboratories, Hercules, CA, USA).

Equal amounts of protein (20 μg) were denatured at 95 °C for 5 min, separated by 12% SDS–PAGE under denaturing conditions, and transferred onto nitrocellulose membranes (Bio-Rad Laboratories, Hercules, CA, USA). Membranes were blocked for 1 h at room temperature in TBS-T (20 mmol/L Tris-HCl [pH 7.6], 137 mmol/L NaCl, and 0.1% Tween-20) containing 5% non-fat dry milk. Following blocking, membranes were washed three times with TBS-T (10 min each) and incubated overnight at 4 °C with the appropriate primary antibodies: rabbit anti-phospho-ERK1/2 (1:1000; Cell Signaling Technology, Danvers, MA, USA) and rabbit anti-glyceraldehyde-3-phosphate dehydrogenase (GAPDH). After incubation, membranes were washed twice with TBS-T (10 min each), followed by one wash with TBS 1X. The membranes were then incubated for one hour with horseradish peroxidase-conjugated goat anti-rabbit IgG secondary antibody (1:2000; Santa Cruz Biotechnology, Santa Cruz, CA, USA). Protein bands were detected using enhanced chemiluminescence (ECL) substrate (SuperSignal, Pierce Biotechnology, Rockford, IL, USA) and visualized using a Bright 1500 imaging system (Invitrogen, Thermo Fisher Scientific, Waltham, MA, USA).

### 4.15. Inhibition of MAPK/ERK Signaling and Assessment of Cell Proliferation and Viability

For each cell line, PC3 WT and EbKD cells were seeded in triplicate wells in 24-well plates. In addition to untreated controls for both WT and EbKD cells, the following treatment conditions were applied: (i) vehicle control (DMSO), (ii) Trametinib inhibitor (10 nM), (iii) exogenous Eb peptide (25 nM), and (iv) a combination of Eb (25 nM) and Trametinib (10 nM). Trametinib (MedChemExpress; Cat. No. HY-10999, Monmouth Junction, NJ, USA) was used as a selective MEK1/2 inhibitor to suppress MAPK/ERK signaling and was prepared in DMSO from a 10 mM stock solution. Each condition was performed in parallel with the presence or absence of UV radiation. Time zero (t = 0) was designated as the time point of UV radiation for all experimental conditions. For UV treatment experiments, cells were pre-treated with the indicated compounds for 1 h prior to UV exposure. Immediately following irradiation, the medium was replaced with fresh medium containing the same treatments, and cells were further incubated to maintain continuous pathway inhibition.

Cell proliferation was assessed at 24 and 48 h post-treatment by harvesting adherent cells via trypsinization and counting them using a Neubauer hemocytometer. Cell viability was evaluated at the same timepoints using the Trypan Blue exclusion assay. Briefly, culture supernatants containing non-adherent (dead) cells were collected, centrifuged, and the resulting pellet was resuspended in a 1:1 mixture of PBS and Trypan Blue. Dead cells were subsequently quantified using the same method. The numbers of viable and non-viable cells were used to calculate proliferation and viability for each condition. Proliferation was expressed as the relative increase in total cell number at 24 and 48 h compared to baseline (t_0_) [% Proliferation = ((cell number_tx_ − cell number_t0_)/cell number_t0_) × 100]. Cell viability was calculated as the percentage of live cells relative to the total cell population [% Viability = (live cells/total cells) × 100].

### 4.16. Proteomic Analysis

Proteomic analysis was performed on PC3 WT and EbKD cells under both normal conditions and following UV irradiation. For each condition, four independent biological replicates were analyzed, and each replicate was measured in triplicate technical runs to ensure analytical reproducibility. For UV-treated samples, protein extraction was carried out 6 h after radiation. Cells were lysed in SDS lysis buffer (4% (*w*/*v*) SDS, 100 mM Tris–HCl (pH 7.6), and 0.1 M dithiothreitol (DTT), with ultrapure water added to the desired final volume). Following lysis, samples were heated at 98 °C for 3 min to ensure protein denaturation. The lysates were subsequently subjected to bath sonication for 5 min to shear genomic DNA and reduce sample viscosity. The extracts were then clarified by centrifugation at 16,000× *g* for 5 min at 4 °C, and the supernatants were collected for downstream proteomic analysis.

For each sample, 30 μL of serum was analyzed. Peptides were separated using an Acclaim PepMap column (75 μm × 50 cm) on an Ultimate 3000 nanoLC system maintained at 40 °C. Chromatographic separation was performed at a flow rate of 350 nL/min using 0.1% formic acid in water as solvent A and 0.1% formic acid in acetonitrile as solvent B. For each injection, 500 ng of peptides were loaded onto the column and eluted using a linear gradient from 8% to 24% solvent B over 50 min, followed by an increase to 36% solvent B over 10 min. The column was subsequently washed with 100% solvent B for 5 min and re-equilibrated at 8% solvent B for 15 min.

Gas-phase fractionation (GPF) of the pooled sample was carried out using 12 windows of 50 *m*/*z* each, covering a range from 400 to 1000 *m*/*z* at 60,000 MS1 with an AGC of 3 × 10^6^ for 60 ms and MS2 at 30,000 with an AGC of 1 × 10^6^ for 60 ms; NCE was set to 27, and +2 was assumed as the default charge state. The GPF-DIA acquisitions used 4 *m*/*z* precursor isolation windows in a staggered window pattern with optimized window placements.

The DIA conditions for sample analysis were as follows: (a) MS1 scans were acquired over 390–1010 *m*/*z* at a resolution of 60,000, with an AGC target of 3 × 10^6^ and an injection time of 60 ms; (b) 76 DIA isolation windows of 8 *m*/*z* each were measured at a resolution of 15,000 in a staggered-window pattern optimized from 400 to 1000 *m*/*z*, using a normalized collision energy (NCE) of 27 and a default charge state of +2.

Raw GPF files were processed with ProteoWizard (v3.0.18299) using 10 ppm overlap demultiplexing after peak picking. Peptide identification was performed with EncyclopeDIA (v0.9) using default settings (10 ppm precursor, fragment, and library tolerances), considering b and y ions with trypsin digestion. The resulting library was imported into Skyline (v20.1) after removing repeated peptides. Raw files were reintegrated using an mProphet model with 1:1 decoys, and peptides were filtered for dotp > 0.7. Total fragment areas for each peptide were summed per protein, and protein abundances were normalized to the total area of each sample. Statistical analysis was carried out in Perseus (v1.6.10.0). Proteins associated with the DNA damage response (DDR) and DNA repair, base excision repair (BER), and nucleotide excision repair (NER) were selected using keyword filtering, and separate heatmaps were generated for each pathway.

### 4.17. Statistical Analysis

All statistical analyses were performed using GraphPad Prism (version 8; GraphPad Software, San Diego, CA, USA) and Microsoft Excel for Microsoft 365 (Microsoft Corp., Redmond, WA, USA). For the exogenous Eb peptide treatment proliferation and viability assays, as well as the rescue and repair experiments, one-way ANOVA followed by Tukey’s post hoc test was performed. However, for the EbKD proliferation and viability assays, as well as the rescue and repair experiments, two-tailed unpaired Student’s t-tests followed by Bonferroni correction were conducted in Excel, while two-way ANOVA followed by Tukey’s post hoc test was performed using Graph Pad, to evaluate the effects of multiple variables. For inhibitor-based experiments, pairwise comparisons between treatment groups were additionally performed using two-tailed unpaired Student’s t-tests with Bonferroni correction to assess the specific effects of Trametinib treatment. All pair-wise comparisons were analyzed in Tukey’s post hoc test, but only the biologically relevant comparisons are highlighted here. Effect sizes were additionally calculated using Hedge’s g, which applies a correction for small sample bias. For the wound healing assay, two-tailed unpaired Student’s t-tests were conducted using Microsoft Excel. For qRT-PCR data, unpaired two-tailed Student’s t-tests were carried out on ΔCt values using Excel. Flow cytometry data was analyzed through two-way ANOVA with Tukey’s post hoc test in Graph Pad. All results are presented as mean average ± standard error (SEM), with a significance set at *p* < 0.05.

### 4.18. Limitations of the Study

Despite the overall coherence of the findings, several limitations should be acknowledged when interpreting the present data. First, although the results suggest that IGF-1Eb/Eb peptide may modulate cellular responses to UV-induced stress, the study remains primarily descriptive and does not fully define the underlying molecular mechanisms involved. In particular, while pharmacological inhibition experiments with Trametinib support the involvement of MAPK/ERK signaling in proliferation-associated responses, they do not establish a direct or exclusive Eb–MAPK/ERK signaling axis.

Second, the observed responses were strongly cell-line-dependent, indicating that the biological effects of the Eb peptide may vary substantially according to tumor type and cellular context. In this respect, proliferation-related and survival-related outcomes should be interpreted separately, since MAPK/ERK inhibition predominantly affected proliferative recovery, whereas viability-related effects were comparatively less pronounced. In addition, although several differences reached statistical significance, some effects, particularly in viability assays, were modest in absolute biological magnitude and should therefore be interpreted cautiously regarding their functional relevance. The relatively small number of biological replicates also reduced statistical power, meaning that certain visually evident differences did not consistently achieve statistical significance.

Methodological limitations may have further influenced viability and apoptosis measurements. Common flow cytometric artifacts include the sensitivity of sub-G0/G1 detection to fixation quality, variability in gating of PI-positive late apoptotic cells, the tendency of intense UV exposure to shift cells toward necrotic phenotypes, and the inherently low early apoptotic fractions, which may have contributed to minor inconsistencies between experimental conditions. Furthermore, in some analyses, relatively elevated effect size estimates may partly reflect sample dispersion and limited sample size.

Finally, although the proteomic analysis identified proteins associated with DNA damage response and repair-related pathways, these observations remain exploratory and do not constitute direct functional validation of DNA repair mechanisms. Additional mechanistic studies incorporating larger sample sizes, complementary molecular approaches, and functional validation assays will therefore be required to more precisely define the contribution of the Eb peptide to cellular stress adaptation and tumor-associated signaling responses.

## 5. Conclusions

Our findings identify IGF-1Eb as a potential stress-adaptive peptide with dual, context-specific functions: lineage-dependent modulation of normal growth and a conserved protective role during UV-induced damage. While its effects under basal conditions varied among the tumor models examined, a more consistent pattern emerged following UV exposure, where Eb depletion was associated with reduced proliferative capacity, increased apoptotic susceptibility, and impaired cellular recovery. Rescue experiments further demonstrated that supplementation with exogenous Eb peptide partially restored proliferation and viability in EbKD cells, supporting a possible functional contribution of this isoform to post-damage adaptation. In addition, signaling analysis revealed modest and transient activation of ERK following Eb exposure, while comparative proteomic profiling identified alterations in multiple proteins associated with DNA damage response pathways. Together, these observations support the possibility that Eb may influence broader cellular stress-response networks rather than acting as a direct regulator of a single pathway. Overall, the data highlights IGF-1Eb as a dynamically regulated IGF-1 isoform whose biological effects appear to depend on cellular context and stress conditions and warrant further mechanistic investigation.

## Figures and Tables

**Table 1:** Primers used in this study.

Gene	Forward	Reverse
***IGF1-Ec***	5′-TATCAGCCCCCATCTACCA-3′	5′-CTTGCGTTCTTCAAATGTACTTCCT-3′
***GAPDH***	5′-CATCACTGCCACCCAGAAGA-3′	5′-TCCACCACCCTGTTGCTGTA-3′

## Data Availability

All the data generated or analyzed during this study are included in this published article.

## Abbreviations

The following abbreviations are used in this manuscript:

IGF	Insulin-like growth factor
IGF-1R	Insulin-like growth factor 1 receptor
IGF-2R	Insulin-like growth factor 2 receptor
IGFBP	Insulin-like growth factor-binding protein
IGF-1Ea/IGF-1Eb/IGF-1Ec	IGF-1 isoforms Ea, Eb, and Ec
EbKD/KD	Eb knockdown/knockdown
WT	Wild type
PEc	Ec-derived peptide
UV	Ultraviolet radiation
BC	Breast cancer
HCC	Hepatocellular carcinoma
MAb	Monoclonal antibody
siRNA	Small interfering RNA
DMEM	Dulbecco’s Modified Eagle Medium
FBS	Fetal bovine serum
PBS	Phosphate-buffered saline
PFA	Paraformaldehyde
FITC	Fluorescein isothiocyanate
DAPI	4′,6-diamidino-2-phenylindole
PI	Propidium iodide
qRT-PCR	Quantitative real-time polymerase chain reaction
cDNA	Complementary DNA
RNA	Ribonucleic acid
ANOVA	Analysis of variance
SD	Standard deviation
ATCC	American Type Culture Collection
TNBC	Triple Negative Breast Cancer
EMT	Mesenchymal transition
